# Supplementary material for: Validating the 2-minute walk test MCID for subacute stroke patients: A Pakistani multicenter cohort analysis
Source: PLoS One. 2026 Apr 15;21(4):e0347056. doi: 10.1371/journal.pone.0347056 (PMC13082714; doi:10.1371/journal.pone.0347056)
Supplement: S1 File — (DOCX) [file pone.0347056.s002.docx]

**STROBE Checklist**

***Determining the Minimally Clinically Important Difference of the 2-Minute Walk Test in Individuals with Subacute Stroke****:* ***A Multicenter Cohort Study****"*

| **Item No.** | **STROBE Recommendation** | **Compliance** | **Details from Manuscript** | **Location in Manuscript** |
| --- | --- | --- | --- | --- |
| **Title and Abstract** |  |  |  |  |
| 1(a) | Indicate the study’s design with a commonly used term in the title or the abstract | Yes | Title includes "Multicenter Cohort Study"; abstract mentions cohort study design. | Title, Abstract |
| 1(b) | Provide in the abstract an informative and balanced summary of what was done and what was found | Yes | Abstract summarizes background, aim, methods (sample size, setting, measures), results (MCID of 33 meters, sensitivity, specificity, correlation), and conclusions. | Abstract |
| **Introduction** |  |  |  |  |
| 2 | Describe the scientific background and rationale for the investigation being reported | Yes | Outlines stroke’s impact on mobility, subacute phase importance, 2MWT role, and gap in MCID knowledge for 2MWT in subacute stroke. | Introduction (Section 1) |
| 3 | State specific objectives, including any prespecified hypotheses | Yes | Aims to determine 2MWT MCID using anchor-based approach; expects MCID to reflect meaningful changes in walking capacity, balance confidence, and functional independence. | Introduction (Section 1, last paragraph) |
| **Methods** |  |  |  |  |
| 4 | Present key elements of study design early in the paper | Yes | Describes prospective, longitudinal cohort study in 10 rehabilitation centers, with trial registration (NCT04892312) and ethical approval. | Methods (Section 2.1) |
| 5 | Describe the setting, locations, and relevant dates, including periods of recruitment, exposure, follow-up, and data collection | Yes | Conducted in 10 Pakistan rehabilitation centers, January 2023–June 2025; assessments at baseline (T0) and post-intervention (T1) after 6–8 weeks. | Methods (Section 2.1) |
| 6(a) | Give the eligibility criteria, and the sources and methods of selection of participants. Describe methods of follow-up | Yes | Inclusion: adults (≥18 years), ischemic/hemorrhagic stroke (<180 days), able to walk. Exclusion: severe cognitive/communication impairments, age <18, mobility-limiting comorbidities. Recruited from inpatient/outpatient centers; follow-up after 6–8 weeks. | Methods (Section 2.2) |
| 6(b) | For matched studies, give matching criteria and number of exposed and unexposed | Not applicable | Not a matched study. | N/A |
| 7 | Clearly define all outcomes, exposures, predictors, potential confounders, and effect modifiers. Give diagnostic criteria, if applicable | Yes | Outcomes: 2MWT (meters), ABC_Gait (0–100), GPE (7-point Likert), MBI. Predictors/confounders: age, sex, stroke severity, baseline 2MWT. Stroke diagnosed per WHO criteria. | Methods (Section 2.4, Data Analysis) |
| 8 | For each variable of interest, give sources of data and details of methods of assessment (measurement). Describe comparability of assessment methods if there is more than one group | Yes | 2MWT: average distance over two trials on 30m walkway. ABC_Gait: gait items from ABC scale. GPE: patient/therapist ratings. MBI: functional independence. Standardized across centers by blinded researchers. | Methods (Section 2.3, 2.4) |
| 9 | Describe any efforts to address potential sources of bias | Yes | Blinded researchers conducted assessments. Multiple imputation handled missing data. Standardized training/protocols ensured consistency. | Methods (Section 2.3, Data Analysis) |
| 10 | Explain how the study size was arrived at | Yes | Sample size of 150 determined for 80% statistical power at 5% significance, larger than prior studies for generalizability. | Methods (Section 2.2) |
| 11 | Explain how quantitative variables were handled in the analyses. If applicable, describe which groupings were chosen and why | Yes | Quantitative variables (2MWT, ABC, ABC_Gait, MBI) analyzed with means, SDs, change scores. ROC curves for MCID cutoff. Logistic regression adjusted for confounders (age, sex, stroke severity, baseline 2MWT). | Methods (Data Analysis) |
| 12(a) | Describe all statistical methods, including those used to control for confounding | Yes | Paired t-tests for pre/post scores. ROC curves for MCID (AUC ≥0.7). Logistic regression adjusted for age, sex, stroke severity, baseline 2MWT. Multiple imputation for missing data. | Methods (Data Analysis) |
| 12(b) | Describe any methods used to examine subgroups and interactions | Yes | Logistic regression examined relationships between 2MWT improvements and baseline variables (e.g., stroke type, assistive device use). | Methods (Data Analysis), Results (Section 3.3) |
| 12(c) | Explain how missing data were addressed | Yes | Multiple imputation used to minimize bias from missing data. | Methods (Data Analysis) |
| 12(d) | If applicable, explain how loss to follow-up was addressed | Partial | Not explicitly reported, but multiple imputation suggests minimal loss to follow-up. | Methods (Data Analysis) |
| 12(e) | Describe any sensitivity analyses | Yes | Distribution-based methods (SEM, effect size) complemented anchor-based MCID calculation. | Results (Section 3.3) |
| **Results** |  |  |  |  |
| 13(a) | Report numbers of individuals at each stage of study—e.g., numbers potentially eligible, examined for eligibility, confirmed eligible, included in the study, completing follow-up, and analysed | Yes | 150 participants enrolled; no explicit mention of loss to follow-up, implying near-complete follow-up. | Results (Section 3.1, Table 1) |
| 13(b) | Give characteristics of study participants (e.g., demographic, clinical, social) and information on exposures and potential confounders | Yes | Table 1: age (64.2 ± 11.8 years), sex (86M/64F), time since stroke (92 ± 46 days), stroke type (68% ischemic), baseline 2MWT (62.5 ± 38.4m), MBI (72.1 ± 19.6), assistive device use (72%). | Results (Section 3.1, Table 1) |
| 13(c) | Indicate number of participants with missing data | Partial | Not explicitly stated, but multiple imputation used, implying minimal missing data impact. | Methods (Data Analysis) |
| 14(a) | Give characteristics of study participants (e.g., demographic, clinical, social) and information on exposures and potential confounders | Yes | Table 2: pre/post means, SDs, changes for 2MWT (36.2 ± 32.8m), ABC (14.6 ± 10.5), ABC_Gait (15.4 ± 11.0), MBI (14.3 ± 9.2), GPE (80% patients, 76.7% therapists improved). | Results (Section 3.2, Table 2) |
| 14(b) | Indicate number of participants with missing data for each variable of interest | Yes | Subgroup data for ischemic vs. hemorrhagic stroke; hemorrhagic group showed greater baseline variability (SD 42.1m vs. 35.8m). | Results (Section 3.1) |
| 15 | Report numbers in each exposure category, or summary measures of exposure | Yes | Outcome measures (2MWT, ABC, ABC_Gait, MBI, GPE) summarized in Table 2 with significant improvements (p < 0.001). | Results (Section 3.2, Table 2) |
| 16(a) | Give unadjusted estimates and, if applicable, confounder-adjusted estimates and their precision (e.g., 95% confidence interval). Make clear which confounders were adjusted for and why they were included | Yes | MCID: 33 meters (80% CI: 30–36m). Logistic regression OR = 1.15 (95% CI: 1.09–1.21) adjusted for age, sex, stroke severity, baseline 2MWT. | Results (Section 3.3) |
| 16(b) | Report category boundaries when continuous variables were categorized | Yes | ABC_Gait improvement ≥10% used as MCID anchor. | Methods (Section 2.4), Results (Section 3.3) |
| 16(c) | If relevant, consider translating estimates of relative risk into absolute risk for meaningful time frame | Not applicable | Study focuses on continuous outcomes (meters walked). | N/A |
| 17 | Report other analyses done—e.g., analyses of subgroups and interactions, and sensitivity analyses | Yes | Subgroup analysis (ischemic vs. hemorrhagic stroke). Distribution-based methods (SEM 9.2m, effect size 0.55) supported anchor-based MCID. | Results (Section 3.3) |
| **Discussion** |  |  |  |  |
| 18 | Summarize key results with reference to study objectives | Yes | MCID of 33 meters established for 2MWT, reflecting meaningful improvements in walking, balance confidence, and functional independence. | Discussion (Section 4) |
| 19 | Discuss limitations of the study, taking into account sources of potential bias or imprecision | Yes | Limitations: potential recall bias in ABC_Gait, focus on subacute stroke limits generalizability, lack of objective anchors (e.g., spasticity). | Discussion (Section 4) |
| 20 | Give a cautious overall interpretation of results considering objectives, limitations, multiplicity of analyses, results from similar studies, and other relevant evidence | Yes | 33-meter MCID is reliable for subacute stroke; compared to 6MWT MCID (50m); suggests future research in chronic stroke and with wearable sensors. | Discussion (Section 4) |
| 21 | Discuss the generalizability (external validity) of the study results | Yes | Multicenter design (10 centers) and n=150 enhance generalizability vs. smaller studies, but limited to subacute stroke. | Discussion (Section 4) |
| **Other Information** |  |  |  |  |
| 22 | Give the source of funding and the role of the funders for the present study and, if applicable, for the original study on which the present article is based | Yes | Funding information provided in the manuscript. | title Page |
