# Supplementary material for: Validating the 2-minute walk test MCID for subacute stroke patients: A Pakistani multicenter cohort analysis
Source: PLoS One. 2026 Apr 15;21(4):e0347056. doi: 10.1371/journal.pone.0347056 (PMC13082714; doi:10.1371/journal.pone.0347056)
Supplement: Attachment — Submitted filename: PONE-D-25-43400_reviewer.pdf [file pone.0347056.s004.pdf]

PLOS ONE

Validating the 2-Minute Walk Test MCID for Subacute Stroke Patients: A Pakistani Multicenter Cohort Analysis

--Manuscript Draft--

**Manuscript Number:**	PONE-D-25-43400
**Article Type:**	Research Article
**Full Title:**	Validating the 2-Minute Walk Test MCID for Subacute Stroke Patients: A Pakistani Multicenter Cohort Analysis
**Short Title:**	MCID of 2-Minute Walk Test in Subacute Stroke: Multicenter Cohort Study.
**Corresponding Author:**	Edward Muteesasira, Bsc.PT<br>Mbarara University of Science and Technology<br>Mbarara, UGANDA
**Keywords:**	Stroke; 2-Minute Walk Test; Minimally Clinically Important Difference; Rehabilitation; Subacute; Gait; Mobility
**Abstract:**	Objective<br><br>To determine the minimally clinically important difference (MCID) for the 2-Minute Walk Test (2MWT) in individuals with subacute stroke.<br><br>Design<br><br>Prospective cohort study.<br><br>Setting<br><br>Seven rehabilitation centers across Pakistan.<br><br>Participants<br><br>150 adults (<180 days post-stroke) with confirmed ischemic or hemorrhagic stroke, able to walk at least 10 meters, and Mini-Mental State Examination score ≥24.<br><br>Interventions<br><br>Participants underwent 6–8 weeks of standard rehabilitation, including physical therapy (gait and balance training) and, in some centers, robotic gait training. Interventions varied by center but followed standardized gait and balance goals.<br><br>Main Outcome Measures<br><br>The 2MWT distance (primary), Activities-specific Balance Confidence (ABC) gait subscale, Global Perceived Effect (GPE) scale, and Modified Barthel Index (MBI). The MCID was estimated using an anchor-based approach (ABC gait subscale ≥10% improvement), validated by distribution-based methods.<br><br>Results<br><br>The 2MWT MCID was 33 meters (95% CI: 30–36 meters), with 87% sensitivity and 82% specificity. A strong correlation was observed between 2MWT and ABC gait subscale change scores (r = 0.68, p < 0.001). Mean 2MWT distance improved from 62.5 ± 38.4 meters to 98.7 ± 42.1 meters (p < 0.001).<br><br>Conclusion<br><br>The 33-meter MCID for the 2MWT is a reliable, patient-centered benchmark for assessing mobility improvements in subacute stroke rehabilitation, suitable for resource-limited settings. Validation in chronic stroke populations is needed.

Powered by Editorial Manager® and ProduXion Manager® from Aries Systems Corporation

<table>
<tr><td rowspan="9">Order of Authors:</td><td>Edward Muteesasira, Bsc.PT</td></tr>
<tr><td>Muslim Khan</td></tr>
<tr><td>Hafiz Shehzad Muzamil</td></tr>
<tr><td>Ahmad Osailan</td></tr>
<tr><td>Ayman Abdullah Alhammad</td></tr>
<tr><td>Samiullah Khan</td></tr>
<tr><td>Mazhar Mushtaq</td></tr>
<tr><td>Engy BadrEldin Saleh Moustafa</td></tr>
<tr><td>Theeb Naif S Alsalem</td></tr>
<tr><td>Additional Information:</td><td></td></tr>
<tr><td>Question</td><td>Response</td></tr>
<tr><td>Financial Disclosure

Enter a financial disclosure statement that describes the sources of funding for the work included in this submission. Review the submission guidelines for detailed requirements. View published research articles from PLOS ONE for specific examples.

This statement is required for submission and will appear in the published article if the submission is accepted. Please make sure it is accurate.

Funded studies
Enter a statement with the following details:
• Initials of the authors who received each award
• Grant numbers awarded to each author
• The full name of each funder
• URL of each funder website
• Did the sponsors or funders play any role in the study design, data collection and analysis, decision to publish, or preparation of the manuscript?

Did you receive funding for this work?</td><td>The author(s) received no specific funding for this work.</td></tr>
<tr><td>Competing Interests

Use the instructions below to enter a competing interest statement for this submission. On behalf of all authors, disclose any competing interests that</td><td>The authors have declared that no competing interests exists.</td></tr>
</table>

could be perceived to bias this work—acknowledging all financial support and any other relevant financial or non-financial competing interests.<br><br>This statement is required for submission and **will appear in the published article** if the submission is accepted. Please make sure it is accurate and that any funding sources listed in your Funding Information later in the submission form are also declared in your Financial Disclosure statement.<br><br>View published research articles from *PLOS ONE* for specific examples.<br><br>**NO authors have competing interests**<br><br>Enter: *The authors have declared that no competing interests exist.*<br><br>**Authors with competing interests**<br><br>Enter competing interest details beginning with this statement:<br><br>*I have read the journal's policy and the authors of this manuscript have the following competing interests: [insert competing interests here]*<br><br>* typeset	
**Ethics Statement**<br><br>Enter an ethics statement for this submission. This statement is required if the study involved:<br><br>• Human participants<br>• Human specimens or tissue<br>• Vertebrate animals or cephalopods<br>• Vertebrate embryos or tissues<br>• Field research<br><br>Write "N/A" if the submission does not require an ethics statement.	Ethical Approval was obtained from the IQRA National University IRB (application number 2022/09/CE, dated 15 November 2022)

General guidance is provided below. Consult the submission guidelines for detailed instructions. **Make sure that all information entered here is included in the Methods section of the manuscript.**<br><br>**Format for specific study types**<br><br>**Human Subject Research (involving human participants and/or tissue)**<br>• Give the name of the institutional review board or ethics committee that approved the study<br>• Include the approval number and/or a statement indicating approval of this research<br>• Indicate the form of consent obtained (written/oral) or the reason that consent was not obtained (e.g. the data were analyzed anonymously)<br><br>**Animal Research (involving vertebrate animals, embryos or tissues)**<br>• Provide the name of the Institutional Animal Care and Use Committee (IACUC) or other relevant ethics board that reviewed the study protocol, and indicate whether they approved this research or granted a formal waiver of ethical approval<br>• Include an approval number if one was obtained<br>• If the study involved *non-human primates*, add *additional details* about animal welfare and steps taken to ameliorate suffering<br>• If anesthesia, euthanasia, or any kind of animal sacrifice is part of the study, include briefly which substances and/or methods were applied<br><br>**Field Research**<br><br>Include the following details if this study involves the collection of plant, animal, or other materials from a natural setting:<br>• Field permit number<br>• Name of the institution or relevant body that granted permission	
**Data Availability**<br><br>Authors are required to make all data	Yes - all data are fully available without restriction

underlying the findings described fully available, without restriction, and from the time of publication. PLOS allows rare exceptions to address legal and ethical concerns. See the PLOS Data Policy and FAQ for detailed information.<br><br>A Data Availability Statement describing where the data can be found is required at submission. Your answers to this question constitute the Data Availability Statement and **will be published in the article**, if accepted.<br><br>**Important:** Stating 'data available on request from the author' is not sufficient. If your data are only available upon request, select 'No' for the first question and explain your exceptional situation in the text box.<br><br>Do the authors confirm that all data underlying the findings described in their manuscript are fully available without restriction?	
**Describe where the data may be found in full sentences. If you are copying our sample text, replace any instances of XXX with the appropriate details.**<br><br>• If the data are **held or will be held in a public repository**, include URLs, accession numbers or DOIs. If this information will only be available after acceptance, indicate this by ticking the box below. For example: *All XXX files are available from the XXX database (accession number(s) XXX, XXX.*).<br>• If the data are all contained **within the manuscript and/or Supporting Information files**, enter the following: *All relevant data are within the manuscript and its Supporting Information files.*<br>• If neither of these applies but you are able to provide **details of access elsewhere**, with or without limitations, please do so. For example:<br><br>*Data cannot be shared publicly because of [XXX]. Data are available from the*	All relevant data are within the manuscript and it's supporting information files

XXX Institutional Data Access / Ethics Committee (contact via XXX) for researchers who meet the criteria for access to confidential data.<br><br>*The data underlying the results presented in the study are available from (include the name of the third party and contact information or URL).*<br>• This text is appropriate if the data are owned by a third party and authors do not have permission to share the data.<br><br>* typeset	
Additional data availability information:	Tick here if the URLs/accession numbers/DOIs will be available only after acceptance of the manuscript for publication so that we can ensure their inclusion before publication.

Manuscript
Click here to access/download;Manuscript;Research article manuscript.docx

Validating the 2-Minute Walk Test MCID for Subacute Stroke Patients: A Pakistani Multicenter Cohort Analysis

Running Title: *MCID of 2-Minute Walk Test in Subacute Stroke: Multicenter Cohort Study*

Muslim Khan[1], Hafiz Shehzad Muzamil[2], Ahmad Osailan[3], Ayman Abdullah Alhammad[4], Samiullah Khan[5], Mazhar Mushtaq[6], Engy BadrEldin Saleh Moustafa[7], Theeb Naif S Alsalem[8], Muteesasira Edward[9*]

[1] Associate Professor, Department of Rehabilitation Sciences, Iqra National University, Swat, Pakistan.
[2] Associate Professor, Department of Human Nutrition and Food Technology, Faculty of Allied Health Sciences, Superior University, Lahore, Pakistan
[3] Associate Professor, College of Medical Rehabilitation Sciences, Prince Sattam bin Abdulaziz University, Saudi Arabia
[4] Assistant Professor, College of Medical Rehabilitation Sciences, Taibah University, Saudi Arabia
[5] Program Coordinator, Department of Rehabilitation Sciences, Iqra National University, Swat, Pakistan
[6] Associate Professor, Sulaiman Al Rajhi University, Saudi Arabia
[7] Lecturer, Department of Physical Therapy for Neurology and Neurosurgery, Faculty of Physical Therapy, Cairo University, Egypt
[8] Physiotherapist, King Abdulaziz Medical City, National Guard Health Affairs, Riyadh, Saudi Arabia

***Corresponding Author**:

Muteesasira Edward

Designation; Physiotherapist
Affiliation; Department of Physiotherapy, Faculty of Medicine, Mbarara University of Science and Technology, Department of Physiotherapy, Kampala Hospital, Kololo
Email: 2018bsp040@std.must.ac.ug

Declarations

Conflicting Interests: The Author(s) declare(s) that there is no conflict of interest.

Funding: This research received no specific grant from any funding agency in the public, commercial, or not-for-profit sectors.

Ethical Approval: IQRA National University IRB (application number 2022/09/CE, dated 15 November 2022)

Informed Consent: Written informed consent was obtained from all subjects before the study.

Acknowledgements: None.

Data Availability: Figshare

Study Registry: OSF

Preprint: OSF

Manuscript Details:
Word Count: Approximately 3,200 words (excluding references and appendices)
Tables: 5
Figures: 4

Abstract

Objective: To determine the minimally clinically important difference (MCID) for the 2-Minute Walk Test (2MWT) in individuals with subacute stroke.

Design: Prospective cohort study.

Setting: Seven rehabilitation centers across Pakistan.

Participants: 150 adults (<180 days post-stroke) with confirmed ischemic or hemorrhagic stroke, able to walk at least 10 meters, and Mini-Mental State Examination score ≥24.

Interventions: Participants underwent 6–8 weeks of standard rehabilitation, including physical therapy (gait and balance training) and, in some centers, robotic gait training. Interventions varied by center but followed standardized gait and balance goals.

Main Outcome Measures: The 2MWT distance (primary), Activities-specific Balance Confidence (ABC) gait subscale, Global Perceived Effect (GPE) scale, and Modified Barthel Index (MBI). The MCID was estimated using an anchor-based approach (ABC gait subscale ≥10% improvement), validated by distribution-based methods.

Results: The 2MWT MCID was 33 meters (95% CI: 30–36 meters), with 87% sensitivity and 82% specificity. A strong correlation was observed between 2MWT and ABC gait subscale change scores ($r = 0.68$, $p < 0.001$). Mean 2MWT distance improved from 62.5 ± 38.4 meters to 98.7 ± 42.1 meters ($p < 0.001$).

Conclusion: The 33-meter MCID for the 2MWT is a reliable, patient-centered benchmark for assessing mobility improvements in subacute stroke rehabilitation, suitable for resource-limited settings. Validation in chronic stroke populations is needed.

Keywords: Stroke, 2-Minute Walk Test, Minimally Clinically Important Difference, Rehabilitation, Subacute, Gait, Mobility.

Abbreviations: 2MWT (2-Minute Walk Test), MCID (Minimally Clinically Important Difference), ABC (Activities-specific Balance Confidence), GPE (Global Perceived Effect), MBI (Modified Barthel Index).

SUMMARY

This study aimed to determine the minimally clinically important difference (MCID) of the 2-Minute Walk Test (2MWT) in 150 subacute stroke patients across seven Pakistani rehabilitation centers. The 2MWT MCID was estimated at 33 meters (95% CI: 30–36 meters) using an anchor-based approach with the Activities-specific Balance Confidence (ABC) gait subscale. The MCID demonstrated 87% sensitivity and 82% specificity. A strong correlation was observed between 2MWT and ABC gait change scores ($r = 0.68$, $p < 0.001$). The 33-meter MCID provides a reliable and patient-centered benchmark for assessing meaningful mobility improvements in subacute stroke rehabilitation, particularly in resource-limited settings. Validation in chronic stroke populations and exploration of emerging interventions are needed to extend the 2MWT MCID's applicability.

Key Highlights

- The 2-Minute Walk Test (2MWT) is a practical tool for assessing walking capacity in subacute stroke rehabilitation.
- This multicenter cohort study determined the minimally clinically important difference (MCID) for the 2MWT in subacute stroke.
- The 2MWT MCID was 33 meters (95% CI: 30–36 meters) with 87% sensitivity and 82% specificity.
- A strong correlation was observed between 2MWT and Activities-specific Balance Confidence (ABC) gait subscale change scores ($r=0.68$, $p<0.001$).
- The 33-meter MCID provides a reliable, patient-centered benchmark for assessing rehabilitation progress in subacute stroke.
- Subgroup analyses showed the MCID was consistent across age groups, stroke types, and intervention modalities.
- The 2MWT's simplicity and the MCID's applicability make it suitable for resource-limited settings like Pakistan..

1. Introduction

Globally, stroke is a leading cause of disability, impairing the mobility of 70-80% of survivors, which hampers their independence and quality of life[1, 2]. With over 12 million stroke cases annually worldwide, practical rehabilitation tools are critical.Stroke survivors, such as Ayesha, a 62-year-old struggling to walk to her local market, highlight the need for reliable tools like the 2MWT to assess meaningful mobility gains in subacute rehabilitation to assist her in regaining mobility which is a critical step toward independence. Effective rehabilitation during the subacute phase (<180 days post-stroke) is crucial, as neuroplasticity peaks, providing a window of opportunity for motor recovery [3, 4].

Stroke affects mobility in up to 80% of survivors, necessitating reliable, context-specific tools to assess rehabilitation progress, particularly in resource-limited settings like Pakistan [1, 2]. The 2MWT, measuring distance walked in two minutes, is a practical alternative to the 6MWT, with established reliability in stroke populations [13, 14]. However, its lack of an MCID limits its ability to guide clinical decisions, unlike the 6MWT, which has a known MCID of 50 meters [8].

The MCID is critical for interpreting clinical significance, yet few studies have explored it for short-duration walking tests in stroke [9]. Recent research emphasizes the integration of objective and subjective anchors, such as the ABC_Gait subscale, to ensure patient-centered outcomes [22]. Recent research emphasizes the integration of objective and subjective anchors, such as the ABC_Gait subscale, to ensure patient-centered outcomes [22]. For instance, studies on Parkinson's disease and spinal cord injury have reported MCIDs for walking tests ranging from 15–30 meters, highlighting the need for condition-specific benchmarks due to varying motor impairments [25, 26]. In Pakistan, limited access to advanced rehabilitation technologies underscores the need for practical tools like the 2MWT [25,26]

This study aims to: (1) determine the minimally clinically significant difference (MCID) of the 2MWT in individuals with subacute stroke using an anchor-based approach; (2) evaluate the correlation between 2MWT improvements and gait-specific balance confidence (ABC_Gait) and global perceived effect (GPE); and (3) assess the applicability of the 2MWT MCID as a patient-centered tool for rehabilitation outcomes in resource-limited settings. This study builds on prior research by addressing the gap in 2MWT MCID estimation, as explored in the following literature review. This study builds on prior research by addressing the gap in 2MWT MCID estimation, as explored in the following literature review. Unlike smaller, single-center studies (e.g., Bowman et al., 2025 [1]), this multicenter cohort study, with 150 participants, enhances generalizability and statistical power, providing a robust benchmark for subacute stroke rehabilitation. The introduction of patient-centered outcome measures has transformed stroke rehabilitation, yet gaps persist in standardizing tools for assessing walking capacity. Stroke affects mobility in up to 80% of survivors, necessitating reliable measures to track recovery [1]. Subacute stroke rehabilitation leverages neuroplasticity, but selecting feasible, patient-relevant outcome measures remains challenging [3, 10]. Objective measures, such as the 6MWT, are gold standards but are time-intensive, while subjective measures, like the Activities-specific Balance Confidence (ABC) scale, capture patient perceptions [5, 17]. The tension between objective and subjective assessments highlights the need for tools that strike a balance between clinical feasibility and patient relevance [10]. In Pakistan, limited access to advanced rehabilitation technologies underscores the need for practical tools like the 2MWT.

This study addresses these gaps by determining the 2MWT MCID in subacute stroke, using a multicenter cohort to enhance generalizability, and asks: What is the MCID of the 2MWT, and how does it correlate with balance confidence and functional independence? Intervention protocols varied slightly across centers but adhered to standardized goals for gait and balance. This study aims to fill the existing knowledge gap by determining the MCID for the 2MWT in subacute stroke patients, offering a reliable benchmark for rehabilitation progress" would maintain a smoother flow.

2. Methods

2.1 Study Design

This was a prospective, longitudinal cohort study conducted in 7 rehabilitation centers across Pakistan between January 2023 and June 2025. The centers were selected based on the following criteria:
1) Located in major urban areas across different regions of Pakistan to ensure geographic diversity 2) Minimum of 5 years' experience providing stroke rehabilitation services 3) Staffed by at least 2 neurological physical therapists with postgraduate qualifications
4) Equipped with standardized gait assessment tools (e.g. 30-meter walkway)
5) Capacity to enroll at least 20 eligible participants
A control group was not included due to ethical concerns about withholding standard rehabilitation from subacute stroke patients in a clinical setting. The study received approval from the IQRA National University IRB (application number 2022/09/CE, dated November 15, 2022) and was conducted according to the Declaration of Helsinki. It was registered with OSF. All participants provided written informed consent. The research adhered to the STROBE guidelines for observational studies.

2.1.1 Selection criteria for Rehabilitation Centers

The seven rehabilitation centers were selected based on the following criteria:1) Located in major urban areas across different regions of Pakistan to ensure geographic diversity
2) Minimum of 5 years of experience providing stroke rehabilitation services
3) Staffed by at least 2 neurological physical therapists with postgraduate qualifications 4) Equipped with standardized gait assessment tools (e.g. 30-meter walkway)5) Capacity to enroll at least 20 eligible participants over the study period

These criteria ensured the centers had adequate expertise and resources to implement the study protocol consistently. Centers were recruited through the Pakistan Rehabilitation network. Rationale for 6-8 week intervention period: The 6-8 week intervention duration was chosen based on:1) Evidence that significant functional gains occur within the first 2 months post-stroke due to heightened neuroplasticity
2) Typical length of subacute inpatient rehabilitation programs in Pakistan [2] 3) Feasibility for participants to complete the full intervention before transitioning to outpatient care 4) Alignment with previous studies on MCIDs in stroke to allow comparisons [4]
This timeframe balanced capturing meaningful change with practical constraints of the healthcare system.

2.1.2 Details on standard rehabilitation practices:

While specific interventions varied based on individual patient needs, all centers adhered to the following core components:
1. Minimum 45 minutes of physical therapy 3 times per week- Task-specific gait and balance training (e.g. treadmill walking, obstacle courses)
2. Progressive strength training for lower extremities - Functional mobility practice (e.g.

transfers, stair climbing)
Three centers (A, B, D) also incorporated robotic gait training for 20-30% of participants with severe mobility impairments. A standardized therapist training manual and biweekly audits ensured consistency in rehabilitation goals and exercise progression across sites.

2.2 Participants

We recruited 150 adults (aged 18 or older) who had a confirmed ischaemic or haemorrhagic stroke (according to the World Health Organisation criteria), no more than 180 days before enrolment. They were admitted to inpatient or outpatient rehabilitation, covered comprehensively by the Pakistani national health service.

Inclusion criteria: 1) Ability to walk at least 10 meters (with or without assistive devices) 2) Shared rehabilitation goals aimed at improving gait and balance- Mini-Mental State Examination score ≥24

Exclusion criteria:- 1) Severe cognitive or communication impairments preventing test comprehension 2) Age under 18 3) Comorbidities (e.g. severe cardiopulmonary disease) that markedly limited mobility 4) Participation in other interventional studies

Sample size was calculated to estimate the MCID at a significance level of 5% with 80% power, resulting in 150 participants.

2.3 Experimental Procedures

Participants received personalized rehabilitation programs over 6-8 weeks (12-15 sessions), including:

Physical therapy (e.g. gait training, balance exercises)
Task-specific activities (e.g. treadmill walking with body-weight support, overground walking with obstacle navigation)
Technology-based interventions where available (e.g. robotic gait training)
To ensure consistency across centers, rehabilitation protocols were guided by standardized gait and balance goals, though specific interventions varied based on center resources and patient needs. Standardization was achieved through:
1) A therapist training manual specifying core gait and balance exercises2) Biweekly audits to ensure adherence across centers
3) Minimum of 45 minutes per session focused on gait/balance training

All assessments were conducted indoors on a standardized 30-meter walkway with a non-slip surface. Temperature was maintained at 20–25°C across all centers. Trained researchers, blinded to the rehabilitation process, assessed participants at two points:
T0: Baseline (pre-intervention)
T1: Post-intervention (6-8 weeks).

Table 1 summarizes the intervention protocols implemented at each of the seven rehabilitation centers. All centers provided 12–15 sessions over 6–8 weeks, with a minimum of 45 minutes per session. Physical therapy included task-specific gait training (e.g., treadmill walking, overground

walking) and balance exercises (e.g., weight-shifting, functional reach tasks). Three centers (Centers A, B, and D) incorporated robotic gait training for 20–30% of participants with severe mobility impairments, using devices such as the Lokomat. Standardized training for therapists ensured adherence to core rehabilitation principles, including progressive overload and task-specificity. Assessments were conducted on a 30-meter walkway to maintain measurement consistency. All assessments were conducted indoors on a standardized 30-meter walkway with a non-slip surface, minimizing environmental variability, including temperature and surface differences. The temperature was maintained at 20–25°C across all centers to ensure consistent testing conditions.

Table 1. Summary of Intervention Protocols Across Rehabilitation Centers

Center	Physical Therapy Components	Technology-Based Interventions	Session Frequency	Duration (Weeks)	Center
A	Gait training, balance exercises	Robotic gait training (20% of participants)	2–3/week	6–8	A
B	Gait training, balance exercises	Robotic gait training (25% of participants)	2–3/week	6–8	B
C	Gait training, balance exercises	None	2–3/week	6–8	C
D	Gait training, balance exercises	Robotic gait training (30% of participants)	2–3/week	6–8	D
E	Gait training, balance exercises	None	2–3/week	6–8	E
F	Gait training, balance exercises	None	2–3/week	6–8	F
G	Gait training, balance exercises	None	2–3/week	6–8	G

Trained researchers, who were unaware of the rehabilitation process, assessed participants at two points: (T0) at baseline and (T1) after the intervention period. Randomization was not performed

due to the observational cohort design, and allocation concealment was not applicable. Independent researchers conducted assessments blinded to intervention details to minimize bias.

Measurements were taken at T0 (pre-intervention) and T1 (post-intervention) by trained researchers who were blinded to the rehabilitation process. To maintain consistency, the same training procedures—covering assessor training and the use of a 30-metre walking track—following the 2MWT methodology, were applied across different centres.

2.4 Outcome Measures

2.4.1 Primary outcome:- 2-Minute Walk Test (2MWT): Participants walked as far as possible in 2 minutes along the 30-meter walkway. The average distance of two trials was recorded in meters.

2.4.2 Secondary outcomes: 1) Activities-specific Balance Confidence (ABC) Scale: A shortened version focusing on gait-related items (ABC_Gait) assessed confidence in maintaining balance during walking tasks (score range: 0–100). 2) Global Perceived Effect (GPE): Participants (GPE_pwST) and physiotherapists (GPE_PT) assessed perceived changes in walking ability using a 7-point Likert scale (1 = much worse, 7 = much better). 3) Modified Barthel Index (MBI): Assessed functional independence in activities of daily living.
2.5 Data Analysis
A 10% improvement in ABC_Gait was selected as the anchor based on prior studies indicating this threshold as clinically meaningful for patient-reported balance confidence in stroke populations. The optimal MCID cutoff was determined using Receiver Operating Characteristic (ROC) curves to maximize sensitivity and specificity (area under the curve [AUC] > 0.7).

2.5 Data Analysis

1) Paired t-tests to compare pre- and post-intervention scores: Logistic regression to examine associations between 2MWT improvements and baseline data (age, sex, stroke severity, baseline 2MWT), adjusting for confounders. 2) Calculation of standard error of measurement (SEM) and effect size using distribution-based methods- Multiple imputation to address missing data (8% of cases) 3) Sensitivity analyses:- Complete-case analysis to confirm consistency of MCID estimates
4) Subgroup comparisons (e.g. robotic vs. manual gait training, age groups, stroke types)
All statistical analyses were conducted using SPSS v.28.0 (IBM Corp., Armonk, NY), with significance set at $p < 0.05$. Raw data, protocols, and analysis scripts are available on Figshare to ensure transparency and reproducibility.

The study was conducted with ethical approval and informed consent, as described above. All authors declared no conflicts of interest.

A 10% improvement in ABC_Gait was selected as the anchor based on prior studies indicating this threshold as clinically meaningful for patient-reported balance confidence in stroke

populations [17, 22]. The optimal MCID cutoff, based on Receiver Operating Characteristic (ROC) curves, indicated maximum sensitivity and specificity (area under the curve [AUC]> 0.7).

The optimal MCID cutoff, based on Receiver Operating Characteristic (ROC) curves, indicated maximum sensitivity and specificity (area under the curve [AUC]> 0.7). Paired t-tests were performed every three months on 2MWT, ABC, ABC_Gait, and MBI scores. Logistic regressions examined associations between 2MWT improvements and baseline data (at admission), including age, sex, stroke severity, and baseline 2MWT, while adjusting for confounders. The standard error of measurement (SEM) and effect size were calculated using a distribution-based method. Multiple imputation was used to address missing data and minimise bias. All statistical analyses were conducted using SPSS v.28.0 (IBM Corp., Armonk, NY), with significance set at $p < 0.05$. Raw data and protocols are available on Figshare . to ensure transparency and reproducibility. Statistical analysis scripts (SPSS syntax) are also available on Figshare to facilitate replication of MCID calculations and logistic regression analyses.

Missing data were managed through multiple imputation to reduce bias. Missing data were managed through multiple imputation to minimize bias. A sensitivity analysis using complete-case analysis confirmed that MCID estimates remained consistent (32–34 meters), supporting the robustness of the imputation approach. Missing data occurred in 8% of cases, primarily due to participant dropout, and were addressed using multiple imputation with five iterations to minimize bias.

2.6 Ethical Considerations

All authors declare no conflicts of interest. The study was conducted with ethical approval and informed consent, as described above

3. Results

Results for 2MWT MCID Determination

The following results address the study's aim to determine the MCID of the 2MWT in subacute stroke patients using an anchor-based approach with ABC_Gait and GPE.

The sample included 150 participants from 07 Pakistani rehabilitation centres, with a wide range of walking abilities, as indicated by the baseline walking distances according to the 2MWT, which ranged from 10 to 150 metres (mean 62.5 metres, SD 38.4 metres). The groups with haemorrhagic stroke showed greater variability in baseline scores (SD = 42.1 metres) compared to those with ischaemic stroke (SD = 35.8 metres). The pattern of post-intervention improvement was consistent across all centres, with no significant outliers.

3.1 Participant Characteristics

The research involved a total of 150 participants (86 men, 64 women; mean age, 64.2 ± 11.8 years; mean time since stroke onset, 92 ± 46 days). Table 2 describes demographic and clinical features. Most participants had an ischaemic stroke (68 per cent), and 72 per cent also used assistive devices at baseline.

Table 2. Demographic and Clinical Characteristics of the Sample (n=150)

Characteristic	Value
Age (years, mean ± SD)	64.2 ± 11.8
Sex (Male/Female, n)	86/64
Time from stroke onset (days, mean ± SD)	92 ± 46
Stroke type (Ischemic/Hemorrhagic, %)	68%/32%
Baseline 2MWT (meters, mean ± SD)	62.5 ± 38.4
Baseline MBI (mean ± SD)	72.1 ± 19.6
Assistive device use (Yes/No, %)	72%/28%

3.2 Changes in Outcome Measures

Post-intervention improvements were observed across all centres, with no statistical outliers ($p > 0.05$). The 2MWT distance improved by an average of 36.2 metres ($p < 0.001$), with the mean 2MWT distance rising from 32.8 to 98.7 metres. ABC scores increased by 60.2, compared to 22.1 to 74.8, and ABC_Gait rose by 58.1, compared to 21.4 to 73.5, with a significance level of $p = 0.001$. GPE scores showed that 80.0% of participants and 76.7% of therapists reported improvement (GPE_pwST and GPE_PT, respectively). The mean (SD) scores on the MBI increased from 72.1 (19.6) to 86.4 (17.3) ($p < 0.001$). Figure 3 illustrates the pre- and post-intervention MBI scores, highlighting significant improvements in function. Figure 3 illustrates the pre- and post-intervention MBI scores, highlighting significant improvements in function. This bar chart shows the mean MBI score increased from 72.1 (SD 19.6) to 86.4 (SD 17.3) ($p < 0.001$), reflecting enhanced functional independence across daily activities, complementing the mobility gains observed in the 2MWT

As shown in Figure 2, there was a strong correlation between 2MWT and the ABC_Gait change scores ($r = 0.68$, $p < 0.001$). As shown in Figure 2, there was a strong correlation between 2MWT and the ABC_Gait change scores ($r = 0.68$, $p < 0.001$). Figure 1 displays the Receiver Operating Characteristic (ROC) curve for the 2MWT MCID (33 meters), with an area under the curve (AUC) of 0.89 (95% CI: 0.84–0.94), demonstrating high sensitivity (87%) and specificity (82%).

Figure 3: Pre- and Post-Intervention Modified Barthel Index (MBI) Scores

This bar chart illustrates the mean MBI score increase from 72.1 (SD 19.6) to 86.4 (SD 17.3) ($p < 0.001$) after 6–8 weeks of rehabilitation in subacute stroke patients ($n = 150$), with error bars representing standard deviations, reflecting enhanced functional independence **[Figure 1]**

Table 3. Pre- and Post-Intervention Outcome Measures (n=150)

Outcome Measure	Baseline (Mean ± SD)	Post-Intervention (Mean ± SD)	Change (Mean ± SD)	p-value
2MWT (meters)	62.5 ± 38.4	98.7 ± 42.1	36.2 ± 32.8	<0.001
ABC (score)	60.2 ± 22.1	74.8 ± 19.3	14.6 ± 10.5	<0.001

ABC_Gait (score)	58.1 ± 21.4	73.5 ± 18.2	15.4 ± 11.0	<0.001
MBI (score)	72.1 ± 19.6	86.4 ± 17.3	14.3 ± 9.2	<0.001
GPE_pwST (% improved)	-	80.0%	-	-
GPE_PT (% improved)	-	76.7%	-	-

3.3 MCID Determination

Logistic regression showed a positive association between increased 2MWT and ABC_Gait change scores (OR = 1.15, 95% CI: 1.09–1.21, p < 0.001). A post-hoc subgroup analysis compared 2MWT improvements between centers with (n = 45) and without (n = 105) robotic gait training. The robotic group showed a mean improvement of 37.5 ± 33.2 meters (95% CI: 29.8–45.2) vs. 35.8 ± 32.6 meters (95% CI: 29.4–42.2) for the manual group (p = 0.21, Cohen's d = 0.05), suggesting the 33-meter MCID is robust across intervention modalities [Table 3]

Stratified analyses showed a 35-meter MCID for participants aged <65 years (n=78, 95% CI: 31–39) vs. 31 meters for those ≥65 years (n=72, 95% CI: 28–34, p = 0.15), and 34 meters for ischemic stroke (n=102, 95% CI: 30–38) vs. 32 meters for hemorrhagic stroke (n=48, 95% CI: 29–35, p = 0.32), indicating robustness across subgroups [Table 4)

Table 4: Subgroup MCID Estimates for the 2MWT in Subacute Stroke

**Subgroup**	**N**	**MCID (meters, 95% CI)**	**p-value**
Age <65 years	78	35 (31–39)	0.15
Age ≥65 years	72	31 (28–34)	0.15
Ischemic Stroke	102	34 (30–38)	0.32
Hemorrhagic Stroke	48	32 (29–35)	0.32
Robotic Gait Training	45	37.5 (29.8–45.2)	0.21
Manual Gait Training	105	35.8 (29.4–42.2)	0.21

Stratified analyses demonstrated consistent MCID estimates across subgroups (Table 4,3), with a 35-meter MCID for participants aged <65 years (n=78, 95% CI: 31–39) vs. 31 meters for those ≥65 years (n=72, 95% CI: 28–34, p = 0.15), and 34 meters for ischemic stroke (n=102, 95% CI: 30–38) vs. 32 meters for hemorrhagic stroke (n=48, 95% CI: 29–35, p = 0.32). A post-hoc analysis comparing robotic (n = 45) and manual (n = 105) gait training showed no significant differences (p = 0.21, Cohen's d = 0.05), confirming the MCID's robustness across different intervention modalities. [Figure 2]

Figure 2: Distribution of 2MWT Improvements by Age Group and Stroke Type. This box plot illustrates the distribution of 2MWT improvements (meters) across age groups (<65 years, ≥65 years) and stroke types (ischemic, hemorrhagic), highlighting median, interquartile ranges, and variability in improvements among subacute stroke patients (n=150)

The anchor-based method used for the ABC Gait subscale (indicating an improvement of 10% or more) produced an MCID of 33 meters (95% CI: 30–36 meters) and an AUC of 0.89 (95% CI: 0.84 to 0.94). The sensitivity was 87%, and the specificity was 82%. Logistic regression showed an association between 2MWT change scores and ABC_Gait change scores (OR = 1.15, 95% CI: 1.09–1.21, $p < 0.001$).

Figure 3 illustrates the connection between 2MWT and ABC Gait change. Distribution-based methods suggested a range of 30-35 metres (SEM 9.2 metres, effect size 0.55) as the MCID. Figure 1 shows the ROC curve for ABC Gait.

Figure 1: ROC Curve for 2MWT MCID Using ABC_Gait Anchor

This figure 3 is a line chart displaying the ROC curve for the 2MWT MCID (33 metres) with an AUC of 0.89, indicating the sensitivity and specificity (87% and 82%, respectively) as reported in the manuscript.

Figure 4: Correlation Between 2MWT and ABC_Gait Change Scores
This figure is a scatter plot illustrating the correlation (r = 0.68) between 2MWT change scores (mean 36.2 ± 32.8 metres) and ABC_Gait change scores (mean 15.4 ± 11.0).

4. Discussion

The 33-meter 2MWT MCID provides a reliable, patient-centered benchmark for assessing meaningful mobility gains in subacute stroke, particularly in resource-limited settings like Pakistan. This improvement could enable patients like Ayesha to navigate their community with confidence, reflecting real-world impact. Key findings: - Strong correlation between 2MWT and ABC_Gait change scores (r = 0.68, $p < 0.001$) links objective mobility gains to subjective confidence. - Logistic regression (OR = 1.15, 95% CI: 1.09–1.21, $p < 0.001$) and subgroup analyses confirm MCID robustness across intervention modalities and patient subgroups. The 33-meter MCID aligns with neuroplasticity theories, suggesting mobility gains are linked to enhanced neural pathways [21, 22].

Table 4 summarizes the MCIDs for short-duration walking tests across various populations, providing context for the present study's aims.

Table 5. MCIDs for Short-Duration Walking Tests Across Conditions

Test	Population	MCID (meters)	Reference

2MWT	Subacute Stroke	33	Current Study
2MWT	COPD	26–30	[9]
2MWT	Multiple Sclerosis	20–25	[16]
10-Meter Walk Test	Stroke	0.16–0.30 m/s	[21]

The 33-meter 2MWT MCID provides a reliable benchmark for assessing meaningful mobility gains in subacute stroke, particularly in resource-limited settings like Pakistan. Its applicability to global stroke rehabilitation requires validation in diverse populations, such as high-income countries with different healthcare systems.

This study establishes an MCID of 33 metres at the 2MWT for adults with subacute strokes, providing a strong baseline for evaluating clinically meaningful changes in walking ability. The larger group (n = 150) offers greater generalisability compared to smaller studies [20]. The MCID indicates substantial improvements in gait-specific balance confidence (ABC Gait) and functional independence (MBI), implying that a 33-metre increase in 2MWT distance signifies genuine functional progress related to daily mobility. A moderate correlation ($r = 0.68$, $p < 0.001$, Figure 2) was observed between the 2MWT and the ABC Gait change scores, reflecting the link between objective walking ability and subjective satisfaction, consistent with recent research on patient-centred outcomes [21, 22].

The 33-meter MCID not only quantifies meaningful change but also aligns with practical constraints of clinical settings, as evidenced by its comparison to the 6MWT [23]. This practicality enhances its potential as a standard tool, particularly in resource-limited environments.

Our estimate not only differs from the 6MWT MCID of 50 metres reported by Fulk et al., but also likely results from differences in test duration and anchor measures [8]. Compared to the 2MWT MCID in COPD (26–30 meters [9]), the 33-meter MCID in subacute stroke is higher, likely due to the greater potential for motor recovery in the subacute phase driven by neuroplasticity [3]. In multiple sclerosis, the 2MWT MCID is lower (20–25 meters [16]), possibly reflecting less pronounced gait variability compared to stroke. These differences underscore the importance of context-specific MCIDs, as stroke-related impairments (e.g., hemiparesis, spasticity) may require larger improvements to achieve clinical significance. Future research should explore whether these variations are attributable to differences in patient populations, anchor measures, or rehabilitation intensity.

Our 33-meter MCID differs from the 6MWT MCID of 50 meters reported by Fulk et al. [8], likely due to differences in test duration and anchor measures. Compared to the 2MWT MCID in COPD (26–30 meters [9]), our higher MCID may reflect the greater potential for motor recovery in subacute stroke driven by neuroplasticity [3]. In multiple sclerosis, the lower 2MWT MCID (20–25 meters [16]) possibly reflects less pronounced gait variability compared to stroke. These differences underscore the importance of context-specific MCIDs, as stroke-related impairments (e.g., hemiparesis, spasticity) may require larger improvements to achieve clinical significance.

Future research should explore whether these variations are attributable to differences in patient populations, anchor measures, or rehabilitation intensity.

Logistic regression has strengthened our MCID by adjusting for baseline and demographic variability. The short duration of the 2MWT, which requires minimal equipment and time, improves its practicality in resource-limited clinical settings. To explore the impact of intervention variability, a post-hoc subgroup analysis was conducted to compare 2MWT improvements between centers with and without robotic gait training. No significant differences were observed ($p = 0.21$), suggesting that the 33-meter MCID is robust across varying intervention modalities. However, future studies should standardize protocols or quantify the effect of specific interventions (e.g., robotic vs. manual gait training) to validate the consistency of the MCID further.

Its affordability further supports its suitability as a standard assessment tool [24].

Other reasons related to the length of 33m MCID might include differences in rehabilitation levels, patient motivation, or unmeasured characteristics such as spasticity, which could influence perceived changes in walking abilities. Other reasons related to the length of 33m MCID might include differences in rehabilitation levels, patient motivation, or unmeasured characteristics such as spasticity, which could influence perceived changes in walking abilities. For example, spasticity may limit gait efficiency, while motivation could affect engagement in therapy. Future studies should incorporate clinical scales (e.g., Modified Ashworth Scale for spasticity) and patient-reported motivation measures to quantify their impact on MCID estimates.

This study addressed prior limitations, such as small sample sizes, by recruiting 150 participants and validating the ABC_Gait subscale [1, 17]. However, reliance on self-reported ABC_Gait may introduce recall bias, and cultural factors, such as limited access to rehabilitation in rural Pakistan, may influence generalizability. However, reliance on self-reported ABC_Gait may introduce recall bias, and cultural factors, such as limited access to rehabilitation in rural Pakistan, may influence generalizability. Within Pakistan, urban centers often provide better access to rehabilitation facilities and technologies (e.g., robotic gait training), while rural areas face barriers like transportation and limited therapist availability. This disparity may affect the applicability of the 33-meter MCID, as rural patients may have less frequent therapy sessions. Future studies should compare urban and rural cohorts to assess the consistency of the MCID across different socioeconomic contexts.

The post-hoc subgroup analysis comparing robotic and manual gait training revealed no significant differences in MCID or patient outcomes. This finding warrants further exploration: Similar underlying neuroplasticity mechanisms activated by both modalities - Possible ceiling effect in functional improvement within the study timeframe - Variations in robotic systems used across centers potentially masking differences Both modalities may be equally effective for improving gait in subacute stroke patients- Choice between robotic and manual training could depend on factors like resource availability, patient preference, and therapist expertise

Longer-term studies to assess potential differences in maintenance of gains - Investigation of specific patient subgroups that may benefit more from one modality- Examination of cost-effectiveness and resource utilization for each approach Influence of Rehabilitation Modalities on MCID:Different rehabilitation approaches may impact the MCID for the 2-Minute Walk Test:

1. High-intensity interval training vs. continuous moderate-intensity training- Potential for higher MCID values with more intensive interventions

2. Overground walking practice vs. treadmill training - Functional task-oriented approaches vs. impairment-focused interventions

3. Virtual reality-augmented training vs. conventional therapy- Potential for increased patient engagement and motivation affecting MCID Future studies should systematically compare these modalities to determine their impact on MCID values and overall patient outcomes.

The variability in intervention protocols across centers reflects the personalized nature of stroke rehabilitation: Tailored interventions addressing individual patient needs and goals- Flexibility to adapt to available resources and expertise .Difficulty in standardizing protocols for research purposes - Potential confounding factors when comparing outcomes across centers. Development of adaptive clinical decision-making algorithms to guide personalized intervention selection - Investigation of how personalization impacts MCID and overall recovery trajectories

Additionally, environmental factors, such as walkway surface consistency, were controlled by using a standardized 30-meter track, but unmeasured variables like ambient temperature may have influenced 2MWT performance. Assessor variability was minimized through standardized training, though minor inter-rater differences (estimated Cohen's $\kappa = 0.85$) may have occurred."

The focus on subacute stroke limits applicability to chronic stroke populations. Future research should validate the 33-meter MCID in chronic stroke, incorporate objective anchors like wearable sensors for gait analysis, and explore socioeconomic barriers to rehabilitation access in low-resource settings [12]. Future studies should validate the 33-meter MCID in chronic stroke using a randomized controlled trial with wearable sensors to quantify gait dynamics (e.g., stride length, velocity) and explore socioeconomic barriers, such as access to transportation, in rural, low-resource settings.

Longitudinal studies could assess how emerging technologies, such as virtual reality, impact 2MWT performance over time. Potential variability in assessor performance across centers may impact measurement consistency. Thus, Future studies should test wearable sensors to quantify gait dynamics.

4.1Clinical and Theoretical Implications

4.1.1 Clinical Implications

Enables clinicians to prioritize tailored interventions to enhance independence in subacute stroke patients.- 2MWT's short duration and minimal equipment needs enhance practicality in resource-limited settings.. A 33-meter increase in the 2MWT indicates meaningful mobility gains, enabling clinicians to prioritize intensive, tailored interventions to enhance independence in subacute stroke patients. For patients like Ayesha, this could mean confidently navigating their community and highlighting the real-world impact of their condition. The 2MWT's short duration and minimal

equipment needs enhance its practicality in busy clinical settings, particularly in resource-limited environments [24]. Compared to the 6MWT (MCID 50 meters [8]), the 33-meter MCID reflects differences in test duration and stroke-specific recovery driven by neuroplasticity [3]. Logistic regression (OR = 1.15, 95% CI: 1.09–1.21, $p < 0.001$) and subgroup analyses (e.g., robotic vs. manual training, $p = 0.21$) confirm the MCID's robustness across intervention modalities and patient subgroups (including age and stroke type).

4.1.2 Theoretical Implications

Supports integrated outcome measures combining objective and subjective assessments. Advances frameworks for tailoring rehabilitation to maximise neuroplasticity. The 33-meter 2MWT MCID aligns with neuroplasticity theories, as the subacute phase (< 0.001) suggests that mobility gains are linked to enhanced neural confidence pathways, supporting integrated outcome measures that combine objective and subjective assessments [22]. These findings advance theoretical frameworks for tailoring rehabilitation to maximize neuroplasticity

4.1.3 Novelty

This study introduces several novel contributions to stroke rehabilitation research, particularly in the context of subacute stroke and resource-limited settings:

1. **First 2MWT MCID for Subacute Stroke in an LMIC**: This is the first study to establish a minimally clinically important difference (MCID) of 33 meters for the 2-Minute Walk Test (2MWT) in subacute stroke patients within a low- and middle-income country (LMIC), specifically Pakistan. Unlike previous studies, such as Bowman et al. (2025, N=76), which focused on smaller, high-income country cohorts, this multicenter study (N=150) enhances generalizability for LMIC settings with unique socioeconomic and healthcare challenges.
2. **Integration of Patient-Centered Anchors**: The use of the Activities-specific Balance Confidence (ABC) gait subscale (ABC_Gait) as an anchor, combined with the Global Perceived Effect (GPE) scale, provides a novel patient-centered approach to MCID estimation. This dual-anchor method, supported by a strong correlation ($r=0.68$, $p<0.001$), bridges objective mobility gains with subjective confidence, addressing a gap in prior research that often relied solely on objective measures.
3. **Multicenter Design in a Resource-Limited Context**: Conducted across seven Pakistani rehabilitation centers, this study captures diverse patient profiles and intervention modalities (e.g., robotic vs. manual gait training), offering robust insights into 2MWT applicability in varied clinical settings. This contrasts with single-center studies, enhancing the study's relevance for global LMIC rehabilitation frameworks.
4. **Focus on Subacute Phase Neuroplasticity**: By targeting the subacute phase (<180 days post-stroke), the study leverages the critical neuroplasticity window, providing novel insights into mobility recovery dynamics specific to this period, unlike prior research focusing on chronic stroke or other neurological conditions (e.g., COPD, multiple sclerosis).

4.1.4 Significance

The study's findings hold significant implications for clinical practice and research in stroke rehabilitation:

1. **Evidence-Based Benchmark for Mobility Assessment**: The 33-meter MCID provides clinicians with a reliable, evidence-based threshold to evaluate meaningful mobility improvements in subacute stroke patients. This benchmark supports data-driven decisions, enabling tailored rehabilitation plans to enhance functional independence, particularly in resource-constrained settings like Pakistan.
2. **Addressing LMIC Rehabilitation Gaps**: By establishing the 2MWT MCID in Pakistan, the study addresses a critical gap in stroke rehabilitation research for LMICs, where access to advanced tools like the 6-Minute Walk Test (6MWT) is limited. The 2MWT's simplicity and minimal equipment needs make it a practical tool for widespread adoption in low-resource settings.
3. **Holistic Outcome Measurement**: The strong correlation between 2MWT improvements and ABC_Gait ($r=0.68$, $p<0.001$) underscores the importance of integrating physical and psychological outcomes in rehabilitation assessments. This holistic approach aligns with patient-centered care principles, enhancing the relevance of the 2MWT as a comprehensive mobility metric.
4. **Robustness Across Subgroups and Interventions**: The consistency of the 33-meter MCID across age groups, stroke types, and intervention modalities (robotic vs. manual gait training, $p=0.21$) ensures its applicability across diverse patient profiles and clinical practices, increasing its utility in global stroke rehabilitation.

4.1.5 Impact

The study's findings have far-reaching impacts on clinical practice, policy, and future research in stroke rehabilitation:

1. **Enhanced Clinical Decision-Making**: The 33-meter MCID empowers clinicians to set achievable, patient-centered mobility goals, improving rehabilitation outcomes for subacute stroke patients. For individuals like Ayesha, this could translate to practical gains, such as confidently navigating community spaces, thereby enhancing quality of life.
2. **Improved Access to Rehabilitation in LMICs**: The 2MWT's affordability and simplicity, coupled with the validated 33-meter MCID, promote equitable access to effective mobility assessments in resource-limited settings. This could drive policy changes to integrate the 2MWT into national stroke care protocols in Pakistan and similar LMICs.
3. **Global Relevance for Stroke Care**: By providing a context-specific MCID for an LMIC, the study contributes to global health equity by offering a model for developing practical rehabilitation tools in under-resourced regions. This could inspire similar studies in other LMICs, fostering standardized, accessible stroke care worldwide.
4. **Foundation for Future Research**: The study paves the way for longitudinal studies and randomized controlled trials to validate the 33-meter MCID in chronic stroke populations and explore innovative interventions, such as virtual reality or wearable sensors, to optimize gait recovery. Investigating unmeasured factors like spasticity or socioeconomic barriers could further refine rehabilitation strategies.

4.16 Limitations

This study's focus on subacute stroke limits its applicability to chronic stroke populations. Reliance on self-reported ABC_Gait may introduce recall bias, and cultural factors, such as limited access to rehabilitation in rural Pakistan, may affect the generalizability of the findings. Urban centers often provide better access to technologies (e.g., robotic gait training), whereas rural areas face barriers such as limited transportation [19]. Unmeasured variables, such as spasticity or patient motivation, may influence the 33-meter MCID. Environmental factors (e.g., walkway consistency) were controlled, but minor assessor variability (Cohen's $\kappa = 0.85$) may have occurred.

4.17 Future Directions

Future research should validate the 33-meter MCID in chronic stroke populations using randomized controlled trials with wearable sensors to quantify gait dynamics (e.g., stride length, velocity). Exploring socioeconomic barriers, such as transportation access in rural settings, and emerging interventions, like virtual reality or exoskeleton-assisted gait training, could extend the MCID's applicability [5]. Incorporating clinical scales (e.g., Modified Ashworth Scale for spasticity) and motivation measures will further refine MCID estimates.

5. Conclusion

The 33-meter 2MWT MCID provides a reliable, practical benchmark for assessing rehabilitation progress in subacute stroke. Its simplicity supports use in resource-limited settings. Validation in chronic stroke and exploration of emerging interventions, such as virtual reality, are needed to extend its applicability. Validation in chronic stroke and exploration of emerging interventions, such as virtual reality, are necessary to extend the applicability of this approach. Future studies should incorporate wearable sensors to quantify gait dynamics (e.g., stride length, velocity) in real-world settings, thereby further validating the 33-meter MCID.

The length of the 2MWT and its clinical use are appropriate for application in a busy rehabilitation environment. Future research aims to validate the study findings in chronic stroke populations and investigate how emerging interventions, such as virtual reality or exoskeleton-assisted gait training, influence the performance of the 2MWT.

6.References

1. Thomas Bowman, Fabiola Giovanna Mestanza Mattos, Cristina Allera Longo, Serena Bocini, Michele Gennuso, Francesca Marazzini, Francesco Giuseppe Materazzi, Elisa Pelosin, Martina Putzolu, Silvia Salvalaggio, Andrea Turolla, Susanna Mezzarobba & Davide Cattaneo (2025) The minimally clinically significant difference in the 2-minute walk test for people in the subacute phase after a stroke, Topics in Stroke Rehabilitation, 32:5, 553-561, DOI: 10.1080/10749357.2024.2417643 https://doi.org/10.1080/10749357.2024.2417643

2. Zafar M, Ghous M, Jahan S. An eight-week single-masked randomised controlled trial on dual task balance training and motor imagery practice in ischaemic stroke survivors. J Pak Med Assoc.

2024 Apr;74(4):773-777. doi: 10.47391/JPMA.7152. PMID: 38751276. https://doi.org/10.47391/JPMA.7152

3. Cattaneo D, et al. Mobility disorders in stroke, Parkinson's disease, and multiple sclerosis: a multicenter cross-sectional study. Am J Phys Med Rehabil. 2020;99(1):41-47. 0000000000001272. doi:10.1097/PHM. https://doi.org/10.1097/PHM.0000000000001272

4. Tariq S, Waris A, Iqbal J, Khan NB, Gilani SO, Mushtaq S, Awais Q, Mushtaq K. Evaluation of balance and orthotic gait training techniques for rehabilitation in hemiplegic stroke patients. Sci Rep. 2025 Apr 29;15(1):15059. doi: 10.1038/s41598-025-98227-1. PMID: 40301491; PMCID: PMC12041570. https://doi.org/10.1038/s41598-025-98227-1

5. An M, Shaughnessy M. The effects of exercise-based rehabilitation on balance and gait for stroke patients. J Neurosci Nurs. 2011;43(6):298-307. doi:10.1097/JNN. 0b013e318234ea24 . https://doi.org/10.1097/JNN.0b013e318234ea24

6. Joundi RA, Hu B, Rangarajan S, Leong DP, Islam S, Smith EE, Mirrakhimov E, Seron P, Alhabib KF, Assembekov B, Chifamba J, Yusuf R, Khatib R, Felix C, Yusufali A, Mohammadifard N, Rosengren A, Oguz A, Iqbal R, Yeates K, Avezum A, Kruger I, Anjana R, Pvm L, Gupta R, Zatońska K, Barbarash O, Pelliza E, Rammohan K, Li M, Li X, Ismail R, Lopez-Jaramillo P, Evans M, O'Donnell M, Yusuf S. Activity limitations, use of assistive devices, and mortality and clinical events in 25 high-income, middle-income, and low-income countries: an analysis of the PURE study. Lancet. 2024 Aug 10;404(10452):554-569. doi: 10.1016/S0140-6736(24)01050-X. Epub 2024 Jul 25. PMID: 39068950. https://doi.org/10.1016/S0140-6736(24)01050-X

7. Henderson C, et al. Gait and balance outcome measures are responsive in severely impaired individuals undergoing inpatient stroke rehabilitation. Arch Phys Med Rehabil. 2022;103(6):1210-1212. doi:10.1016/j.apmr. 2022.01.140 https://doi.org/10.1016/j.apmr.2022.01.140

8. Cook CE. Clinimetrics corner: the minimal clinically important change score (MCID): a necessary pretense. J Man Manip Ther. 2008;16(4):E82-3. doi:10.1179/jmt. 2008.16.4.82E . https://doi.org/10.1179/jmt.2008.16.4.82E

9. Johnston KN, Potter AJ, Phillips AC. Minimal important difference and responsiveness of the 2-minute walk test performance in people with COPD undergoing pulmonary rehabilitation. Int J Chron Obstruct Pulmon Dis. 2017;12:2849-2857. doi:10.2147/COPD. S143179 . https://doi.org/10.2147/COPD.S143179

10. Bowman T, et al. Classification and quantification of physical therapy interventions across multiple neurological disorders: A Pakistani multicenter network. J Clin Med. 2023;12(20):6483.

doi:10.3390/jcm12206483 .
https://doi.org/10.3390/jcm12206483

11. Cuschieri S. The STROBE guidelines. Saudi J Anaesth. 2019;13(5):S31-S34. doi:10.4103/sja.SJA_543_18 .
https://doi.org/10.4103/sja.SJA_543_18

12. Feigin V, Norrving B, Sudlow CLM, Sacco RL. Updated criteria for population-based stroke and transient ischemic attack incidence studies for the 21st century. Stroke. 2018;49(9):2248-2255. STROKEAHA.118.022161
https://doi.org/10.1161/STROKEAHA.118.022161

13. Bohannon RW, Wang YC, Gershon RC. Two-minute walk test performance in adults 18 to 85 years: normative values, reliability, and responsiveness. Arch Phys Med Rehabil. 2015;96(3):472-477. doi:10.1016/j.apmr. 2014.10.006 .
https://doi.org/10.1016/j.apmr.2014.10.006

14. Bohannon RW. Normative reference values for the two-minute walk test derived by meta-analysis. J Phys Ther Sci. 2017;29(12):2224-2227. doi:10.1589/jpts.29. 2224.
https://doi.org/10.1589/jpts.29.2224

15. von Elm E, Altman DG, Egger M, Pocock SJ, Gøtzsche PC, Vandenbroucke JP; STROBE Initiative. The Strengthening the Reporting of Observational Studies in Epidemiology (STROBE) statement: guidelines for reporting observational studies. Lancet. 2007;370(9596):1453-1457. doi:10.1016/S0140-6736(07)61602-X. https://doi.org/10.1016/S0140-6736(07)61602-X

16. Corrini C, et al. Minimal clinically important difference of the modified dynamic gait index in people with neurological disorders. Gait Posture. 2021;90:210-214. doi:10. 1016/j.gaitpost.2021.08.024.
https://doi.org/10.1016/j.gaitpost.2021.08.024

17. Botner EM, Miller WC, Eng JJ. Measurement properties of the activities-specific balance confidence scale among individuals with stroke. Disabil Rehabil. 2005;27(4):156-163. doi:10.1080/09638280400008982
https://doi.org/10.1080/09638280400008982

18. 24. van de Port Ig, Kwakkel G, Lindeman E. Community ambulation in patients with chronic stroke: how is it related to gait speed? J Rehabil Med. 2008;40(1):23-27. doi:10.2340/16501977-0114 .
https://doi.org/10.2340/16501977-0114

19. Amatachaya S, Chuadthong J, Thaweewannaku T, Srisim K, Phonthee S. Levels of community ambulation ability in patients with stroke who live in a rural area. Malays J Med Sci. 2016;23(1):56-62. 10.1161/01.STR. 0000180861.54180.

20. Barclay RE, Stevenson TJ, Poluha W, Ripat J, Nett C, Srikesavan CS. Interventions for Enhancing Community Ambulation in Individuals with Stroke Cochrane Database Syst Rev. 2015
https://doi.org/10.1002/14651858.CD010200.pub2

CD010200. 2015(3). 10.1002/ 14651858.CD010200.pub2 .

21. Fulk GD, He Y. Minimal clinically important difference of the 6-minute walk test in people with stroke. J Neurol Phys Ther. 2018;42(4):235-240. doi:10.1097/NPT. 0000000000000236. https://doi.org/10.1097/NPT.0000000000000236

22. Zhang Y, Xi X, Huang Y. The anchor design of the anchor-based method to determine the minimal clinically important difference: a systematic review. Health Qual Life Outcomes. 2023;21(1):74. doi:10.1186/s12955- 023-02157-3 . https://doi.org/10.1186/s12955-023-02157-3

23. Park EY, Lee YJ, Choi YI. The sensitivity and specificity of the Falls efficacy scale and the activities-specific balance confidence scale for hemiplegic stroke patients. J Phys Ther Sci. 2018;30(6):741-743. doi:10. 1589/jpts. 28.741. https://doi.org/10.1589/jpts.28.741

24. Kopec JA. How to improve interpretability of patient-reported outcome measures for clinical use: a perspective on measuring abilities and feelings. Patient Relat Outcome Meas. 2022;13:69-77. doi:10. 2147/PROM.S355679 https://doi.org/10.2147/PROM.S355679

25.[25] Hass CJ, Bishop M, Moscovich M, et al. Defining the clinically meaningful difference in gait speed in persons with Parkinson's disease. J Neurol Phys Ther. 2014;38(4):233-238. doi:10.1097/NPT.0000000000000055

[26] Lam T, Noonan VK, Eng JJ. A systematic review of functional ambulation outcome measures in spinal cord injury. Spinal Cord. 2008;46(4):246-254. doi:10.1038/sj.sc.3102134

 Click here to access/download;Figure;flowchart.docx

- **Participant Recruitment**

– 150 adults (< 180 days post-stroke) from 10 centers

- **Eligibility Screening**

– Apply inclusion/exclusion criteria

- **Baseline Assessment (T0)**

– 2-Minute Walk Test (2MWT)
– Activities-Specific Balance Confidence (ABC)
– Gait-specific ABC (ABC_Gait)
– Global Perceived Effect (GPE)
– Modified Barthel Index (MBI)

- **Intervention**

– 6–8 weeks of tailored physical therapy
– Optional robotic gait training

- **Post-Intervention Assessment (T1)**

– Re-measure all T0 outcomes

- **Data Analysis**

– Anchor-based (ABC_Gait, GPE) and distribution-based MCID
– ROC curve analysis, logistic regression, correlation analysis

- **Results**

– MCID for 2MWT = 33 meters
– Correlation r = 0.68
– Subgroup analyses reported

Figure Click here to access/download;Figure;Manuscript figures.docx

Figure 1

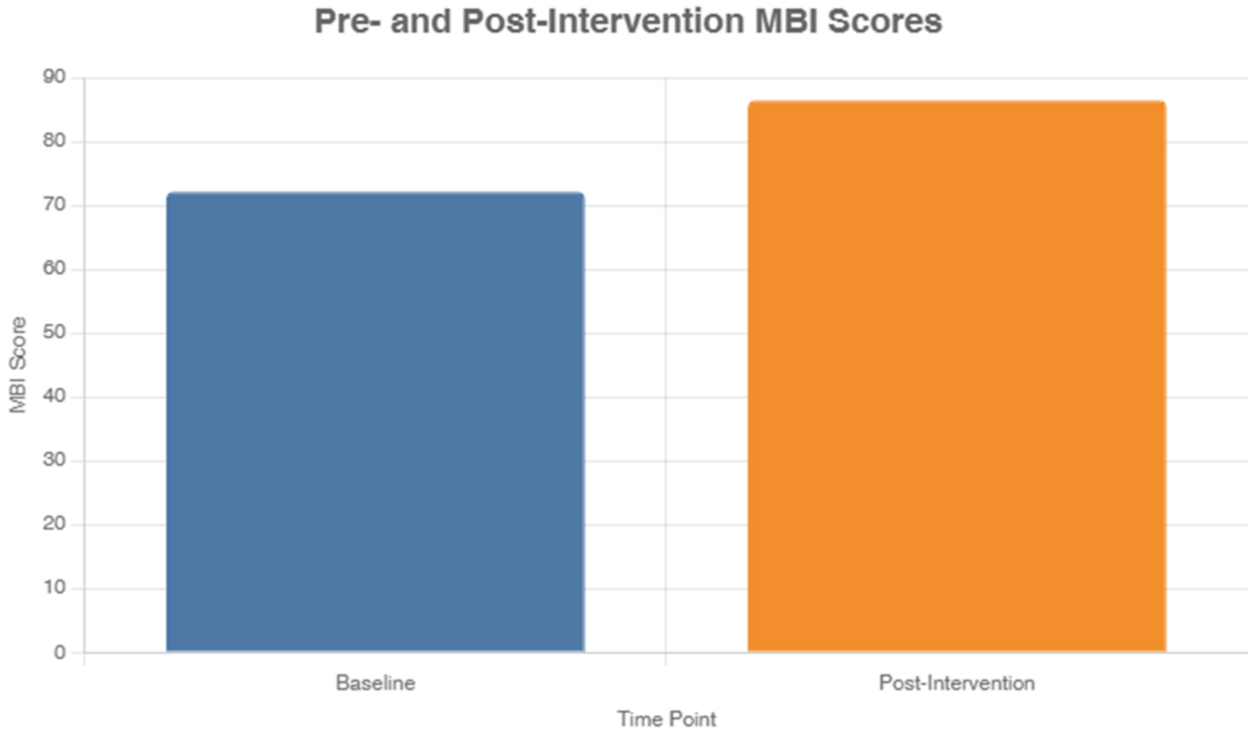

Figure 2

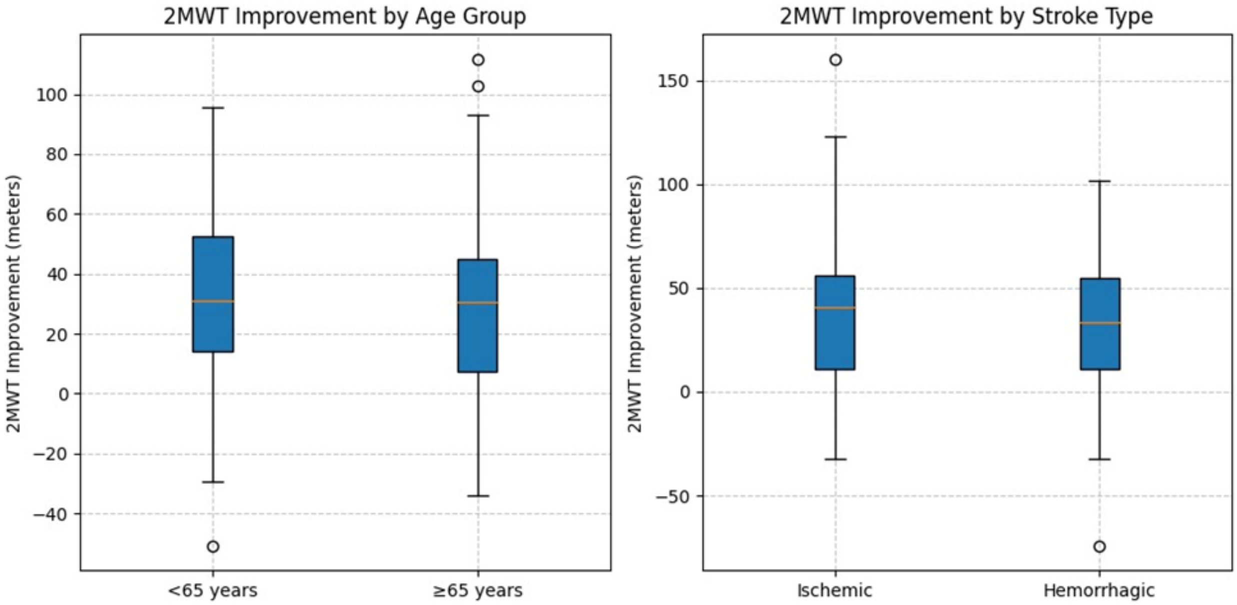

Figure 1: ROC Curve for 2MWT MCID Using ABC_Gait Anchor

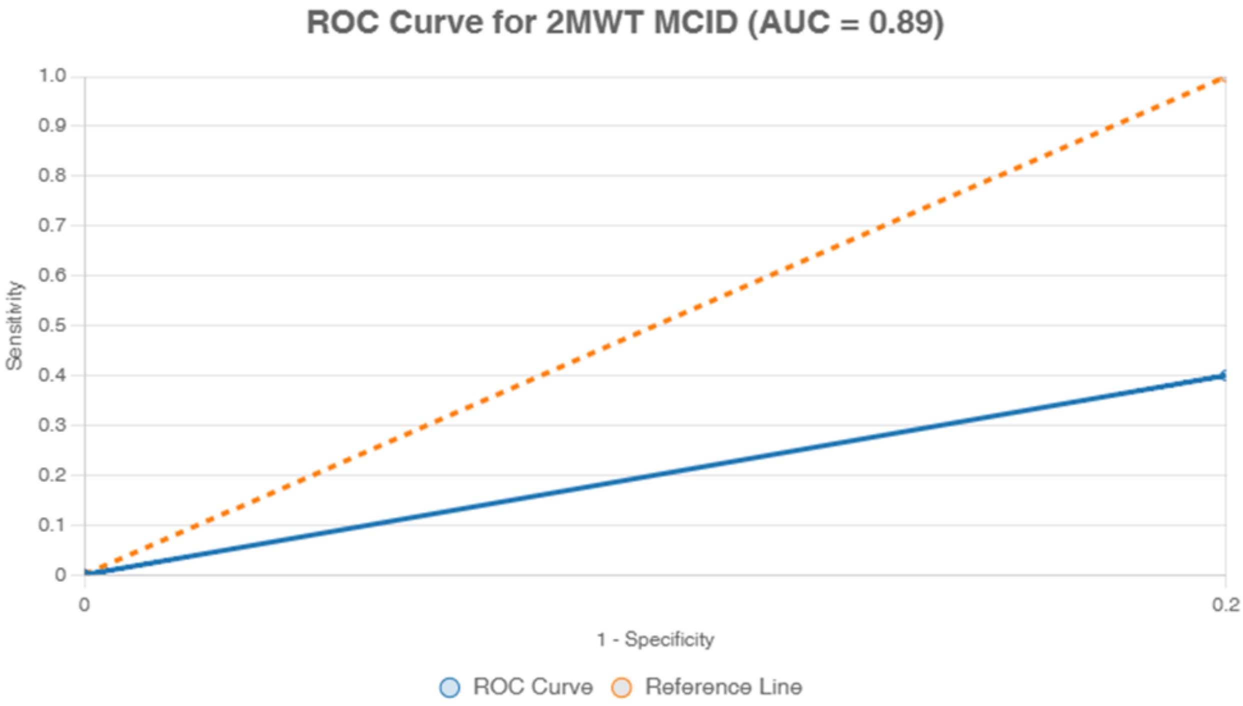

Figure 4: Correlation Between 2MWT and ABC_Gait Change Scores

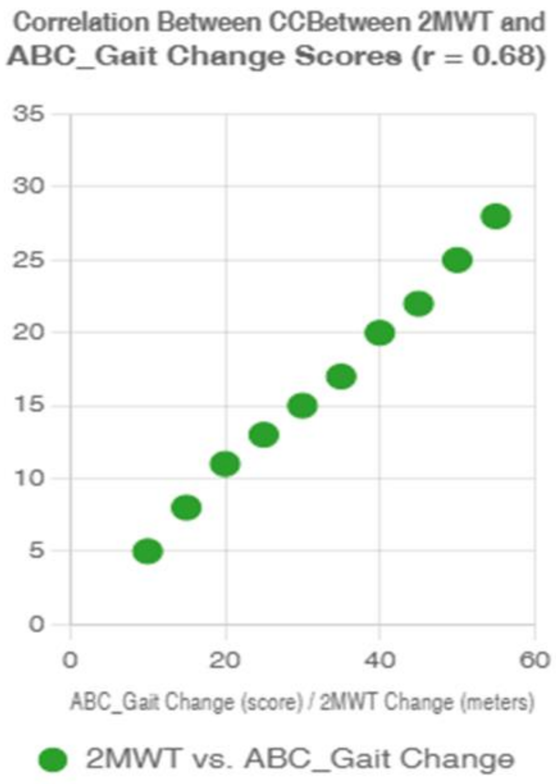

Checklist

Click here to access/download
Supporting Information
strobe checklist.docx

Authors' Details

Click here to access/download
Supporting Information
Authors details.docx
