# Supplementary material for: Validating the 2-minute walk test MCID for subacute stroke patients: A Pakistani multicenter cohort analysis
Source: PLoS One. 2026 Apr 15;21(4):e0347056. doi: 10.1371/journal.pone.0347056 (PMC13082714; doi:10.1371/journal.pone.0347056)
Supplement: Attachment — Submitted filename: Overall Evaluation.docx [file pone.0347056.s003.docx]

**Overall Evaluation**

This manuscript presents a multicenter cohort study evaluating the minimally clinically important difference (MCID) for the 2-Minute Walk Test (2MWT) in subacute stroke patients across seven rehabilitation centers in Pakistan. The topic is clinically relevant, especially for low- and middle-income settings, and the study adds valuable evidence to an underexplored area. The large sample size (n=150) and the combined use of anchor-based and distribution-based approaches strengthen the methodological rigor. The estimated MCID of 33 meters is well supported by the analyses.

While the study has several strengths, the manuscript in its current form is substantially longer than necessary and suffers from repetition, structural inconsistencies, and organizational issues that limit clarity and readability. A focused revision is recommended before the manuscript can be considered for publication.

**Major Strengths**

1. **Multicenter design** across seven facilities enhances generalizability.
2. **Robust methodology**, including both anchor-based and distribution-based MCID estimation.
3. **Comprehensive statistical analysis**, including ROC curves, logistic regression, and subgroup comparisons.
4. **Clinically relevant findings** aligned with patient-centered outcome measurement.
5. **Meaningful contribution** to rehabilitation research in LMIC contexts.

**Major Concerns**

**1. Repetition Throughout the Manuscript**

Significant portions of text—particularly in the Introduction, Methods, Data Analysis, and Discussion—are repeated verbatim or conceptually. This affects readability and dilutes key messages.

**Recommendation:** Remove redundant sections and streamline content.

**2. Introduction Lacks Focus**

The introduction includes multiple narrative threads, duplicated rationale for MCID, and repeated literature background.

**Recommendation:** Condense into four clear paragraphs:
(1) stroke burden and mobility impairment;
(2) role of the 2MWT;
(3) need for MCID, especially in LMICs;
(4) specific study aim.

**3. Methods Section Requires Reorganization**

The Methods contain duplicated material (center selection, intervention protocols, and statistical descriptions) and mix narrative with bullet points.

**Recommendation:** Reorganize into standardized subsections:

- Study Design
- Setting and Centers
- Participants
- Interventions
- Outcome Measures
- Data Collection
- Statistical Analysis

**4. Inconsistent Tables and Figures**

Figure numbering is duplicated, some tables are described twice, and some figures lack proper captions.

**Recommendation:** Standardize tables and figures, ensuring each appears once and is clearly labeled.

**5. Discussion Is Overly Long and Repetitive**

The Discussion restates points already addressed in the Introduction and Results and includes broad, non-specific statements.

**Recommendation:** Reframe into four concise paragraphs:
summary of findings, comparison with prior studies, strengths/limitations, and implications/future directions.

**6. Language and Style Issues**

The manuscript includes long sentences, occasional informal language, and inconsistent tense usage.

**Recommendation:** Edit for clarity, conciseness, and scientific tone.

**7. Ethical and Administrative Information Repeated**

Funding, conflict of interest, and ethics approval statements appear more than once.

**Recommendation:** Present these details only in the designated section.

**Minor Comments**

- Include a CONSORT/STROBE flow diagram.
- Clarify the method used to address missing data.
- State whether assessors received standardized training or inter-rater reliability testing.
- Provide the sample size calculation formula.
- Offer a practical interpretation of the 33-m MCID (e.g., approximate steps or gait speed difference).

**Recommendation: Major Revision**

The study is scientifically sound and clinically important. With substantial revision focusing on structure, clarity, and removal of redundancy, the manuscript has strong potential for publication.
