# Supplementary material for: Validating the 2-minute walk test MCID for subacute stroke patients: A Pakistani multicenter cohort analysis
Source: PLoS One. 2026 Apr 15;21(4):e0347056. doi: 10.1371/journal.pone.0347056 (PMC13082714; doi:10.1371/journal.pone.0347056)
Supplement: Attachment — Submitted filename: Response to Reviewers.docx [file pone.0347056.s005.docx]

**Manuscript reference:** [PONE-D-25-43400] - [EMID: b8822de2989178e8]

**Decision:** Major Revision

**Title:** Validating the 2-minute walk test MCID for subacute stroke patients: A Pakistani multicenter cohort analysis.

**PLOS ONE,**

***Dear Editor,***

We appreciate your oversight of our manuscript and the opportunity to address the insightful comments from the respective reviewers. We extend our sincere gratitude to the reviewers for their valuable feedback which is aimed at improving our manuscript.

Please find below our point-by-point response to all the issues raised by the reviewers. Our responses below list the reviewers’ comments followed by a description of the necessary amendments we the authors have made in bold.

The page numbers of the responses to reviewer comments correspond to the clean version of the revised manuscript.

We look forward to hearing from you at your earliest convenience.

Kindly,

Edward Muteesasira.

**Editor’s Comments**

**Journal Requirements:**

When submitting your revision, we need you to address these additional requirements.

1. Please ensure that your manuscript meets PLOS ONE's style requirements, including those for file naming.

**Response: The manuscript has been edited and structured to conform to PLOS ONE’s style requirements, including those for file naming.**

2. Please include a complete copy of PLOS’ questionnaire on inclusivity in global research in your revised manuscript.

**Response: We have included a complete copy of PLOS’ questionnaire on inclusivity in global research as part of the submitted files under support documents to accompany the revised manuscript.**

3. We note that your Data Availability Statement is currently as follows; “All relevant data are within the manuscript and it's supporting information files” Please confirm at this time whether or not your submission contains all raw data required to replicate the results of your study. Authors must share the “minimal data set” for their submission. PLOS defines the minimal data set to consist of the data required to replicate all study findings reported in the article, as well as related metadata and methods.

**Response: The authors confirm that all relevant data are within the manuscript and it’s supporting information files.**

4. Your ethics statement should only appear in the Methods section of your manuscript. If your ethics statement is written in any section besides the Methods, please move it to the Methods section and delete it from any other section. Please ensure that your ethics statement is included in your manuscript, as the ethics statement entered into the online submission form will not be published alongside your manuscript.

**Response: The ethics statement has been placed under the method section of the revised manuscript and any repetitions elsewhere have been deleted.**

5. Please include a caption for figures 2 and 3.

**Response: We have included captions for figures 2 and 3 in the revised manuscript.**

6. We note you have included a table to which you do not refer in the text of your manuscript. Please ensure that you refer to Table 5 in your text; if accepted, production will need this reference to link the reader to the Table.

**Response: We have linked Table 5 to the respective text in the revised manuscript.**

7. If the reviewer comments include a recommendation to cite specific previously published works, please review and evaluate these publications to determine whether they are relevant and should be cited. There is no requirement to cite these works unless the editor has indicated otherwise.

**Response: We have included appropriate references to Reviewer #1 comment 2(The selection of the ABC_Gait subscale (≥10% improvement) as the anchor is appropriate but should be better justified with references supporting its clinical meaningfulness in stroke populations.) in the revised manuscript.**

**Reviewer #1**

***Dear Authors, I would like to thank the authors for submitting the manuscript entitled “Validating the 2-Minute Walk Test MCID for Subacute Stroke Patients: A Pakistani Multicenter Cohort Analysis.” The study addresses an important gap in stroke rehabilitation research by determining the minimally clinically important difference (MCID) for the 2-Minute Walk Test (2MWT) among subacute stroke patients in a developing country context. The multicenter approach, adequate sample size, and emphasis on patient-centered outcomes are commendable and add value to the literature on functional recovery metrics after stroke. However, to improve its scholarly value and readability, significant revisions are required for the following points :***

1-Clarity and Conciseness of Introduction

The introduction provides a good rationale for the study but includes several redundant sentences (e.g., repeated statements about neuroplasticity and the need for context-specific tools).

Consider streamlining paragraphs to maintain focus on the knowledge gap: Why the 2MWT MCID has not been established for subacute stroke patients, and why this is particularly relevant for Pakistan.

**Response: We have removed redundant statements within the introduction section and have streamlined the introduction section in four clear paragraphs; with the first paragraph addressing stroke burden and importance of mobility, the second one addressing the role and relevance of the 2MWT,the third one addressing the lack of MCID evidence, especially in LMICs and the last paragraph concluding with the precise study aim.**

2-Rationale for Anchor Selection

The selection of the ABC_Gait subscale (≥10% improvement) as the anchor is appropriate but should be better justified with references supporting its clinical meaningfulness in stroke populations.

**Response: We have included two references [17,22] to justify our selection of the ABC_Gait subscale (≥10% improvement) as the anchor in the revised manuscript. This is clearly stated on line 176-178 of the clean copy of the revised manuscript.**

Please explain why the Global Perceived Effect (GPE) was not used as the primary anchor, given its patient-centered nature.

**Response: While recognizing the fact that the Global Perceived Effect (GPE) is a patient centered outcome measure, its methodological limitations particularly its susceptibility to recall bias. Our study required us to assess participants at two points; T0: Baseline (pre-intervention) and T1: Post-intervention (6-8 weeks), we would have faced significant limitations with recall bias especially in the follow-up phase (post-intervention assessment). In order to minimize the risk of recall bias, we utilized the ABC_Gait subscale (≥10% improvement) as the anchor.**

3-Statistical Analysis Details

The manuscript mentions paired t-tests, logistic regression, and ROC analysis, but the criteria for choosing these models and assumptions testing (e.g., normality) are not clearly described.

**Response: The authors have added “According to the criteria of normality, we utilized;(i) paired t-tests to compare pre- and post-intervention scores, logistic regression to examine associations between 2MWT improvements and baseline data (age, sex, stroke severity, baseline 2MWT) as well as adjusting for confounders, (ii) calculation of standard error of measurement (SEM) and effect size using distribution-based methods. This is stated under the analysis section of the clean copy of the revised manuscript(Line 167-171)**

Clarify how missing data (8%) were handled and how multiple imputation may have affected the robustness of the MCID estimates.

**Response:** **Missing data occurred in 8% of cases, primarily due to participant dropout, and were addressed using multiple imputation with five iterations to minimize bias. (Line 182-184 of the clean copy of the revised manuscript). A sensitivity analysis using complete-case analysis confirmed that MCID estimates remained consistent (32–34 meters) supporting the robustness of the imputation approach. (Line 181-182 of the clean copy of the revised manuscript)**

Indicate the AUC confidence intervals in the Results section and interpret what an AUC of 0.89 means in practical clinical terms.

**Response: We have indicated the AUC confidence intervals in the results section of the clean copy of the revised manuscript (Line 255). The AUC of 0.89 demonstrates a high sensitivity (87%) and specificity (82%) of the 2MWT MCID estimate. (Line 221-222 of the clean copy of the revised manuscript).**

4-Interpretation of MCID Value

The MCID of 33 meters is statistically sound, but the discussion should better contextualize this value:
How does it compare to the 6MWT MCID (≈50 m) and other 2MWT MCIDs in neurological conditions?

**Response: This is addressed under the discussion section; Table 5 which summarizes the comparisons with other populations, and from Line 274-284 of the clean copy of the revised manuscript which states, The 33-meter MCID not only quantifies meaningful change but also aligns with practical constraints of clinical settings, as evidenced by its comparison to the 6MWT [23]. This practicality enhances its potential as a standard tool, particularly in resource-limited environments. Our estimate not only differs from the 6MWT MCID of 50 meters reported by Fulk et al., but also likely results from differences in test duration and anchor measures [8]. Compared to the 2MWT MCID in COPD (26–30 meters [9]), the 33-meter MCID in subacute stroke is higher, likely due to the greater potential for motor recovery in the subacute phase driven by neuroplasticity [3]. In multiple sclerosis, the 2MWT MCID is lower (20–25 meters [16]), possibly reflecting less pronounced gait variability compared to stroke. These differences underscore the importance of context-specific MCIDs, as stroke-related impairments (e.g., hemiparesis, spasticity) may require larger improvements to achieve clinical significance.**

What does a 33-meter improvement represent in functional independence or patient quality of life?
Consider adding a short paragraph linking this finding to functional thresholds (e.g., community ambulation, household walking).

**Response: We have added a short paragraph under the discussion section on line 303-308 that states, “****The 33-meter MCID improvement represents a meaningful improvement in the overall patient’s functional exercise capacity because it enhances the stroke patient’s ability to perform basic mobility tasks efficiently and independently within his/her home environment. It’s also worth noting that achieving a greater walking distance is associated with an improved functional capability to move around within the community thus leading to overall independence in the performance of the activities of daily living (ADLs) [18,28]”**

5-Subgroup and Sensitivity Analyses

The robustness of MCID across subgroups (age, stroke type, robotic vs. manual training) is well presented, but interpretation could be more clinically oriented.
For example: explain why MCID differences by age (35 m vs. 31 m) are not statistically significant yet may hold clinical implications for rehabilitation intensity planning.

**Response: We state on line 233-234 that “35-meter MCID for participants aged <65 years (n=78, 95% CI: 31–39) vs. 31 meters for those ≥65 years (n=72, 95% CI: 28–34, p = 0.15)” ,Much as this is not statistically significant possibly arising from our data variability, test duration and anchor measurements, this however does not negate its clinical significance for rehabilitation planning.**

6-Figures and Tables

Figures 1–4 and Tables 3–4 contain valuable data, but legends are overly descriptive and sometimes repetitive.

Simplify figure captions to highlight key findings (e.g., “ROC curve showing optimal 33 m cutoff for 2MWT MCID with 87% sensitivity and 82% specificity”).

**Response: We have reduced the wording of the figure/Table legends and repetitions have been deleted accordingly.**

Ensure that all figures are labeled consistently and referenced appropriately in the text.

**Response: All figures and tables have been labeled consistently and referenced appropriately.**

7-Discussion and Conclusion

The discussion effectively summarizes the results but could benefit from a clearer “clinical implications” section, specifying how rehabilitation clinicians in resource-limited settings can use the 33 m MCID threshold for decision-making.

**Response: The Discussion section has been streamlined in line with the following four aspects with each representing a corresponding paragraph. (i) Key findings, (ii) Comparison with existing literature, (iii) Strengths and limitations and lastly, (iv) Implications and future research**

The conclusion could be more concise, emphasizing future directions such as validation in chronic stroke and longitudinal functional outcomes.

**Response: conclusion section has been streamlined as recommended above.**


8-Language and Style

The manuscript would benefit from a light language edit to remove redundancies and improve fluency (e.g., avoid repeating “reliable benchmark for assessing mobility improvements” multiple times).

**Response: All redundant statements and repetitions have been removed throughout the manuscript and proof reading done to rule out any grammatical inconsistencies.**

Replace informal phrases like “patients such as Ayesha” with more neutral examples or remove for conciseness.

**Response: Informal phrases like “patients such as Ayesha” have been removed and replaced with more neutral examples for purposes of conciseness.**

9-Formatting

Ensure uniform citation formatting and numbering consistency.

**Response: This has been adhered to throughout the clean copy of the revised manuscript.**

Define abbreviations (e.g., ABC_Gait, GPE) at first mention in both abstract and text.

**Response: All abbreviations have been clearly defined at their first mention throughout the manuscript.**

I encourage you to revise the manuscript thoroughly in response to the comments above. I look forward to reviewing the improved version

**Response: The manuscript has been substantially revised in light of the above comments.**

**Many Thanks for those comments aimed at improving the scholarly power and readership of our manuscript.**

**Reviewer #2**

***Thank you for submitting this important and clinically relevant manuscript. The study addresses a meaningful gap by establishing an MCID for the 2-Minute Walk Test in subacute stroke patients within a multicenter LMIC context. The research question is valuable, the methodology is generally sound, and the findings have potential impact for rehabilitation practice. However, the manuscript requires significant revision to improve clarity, structure, and readability. My detailed comments are below.***

**Major Comments**
1. Repetition across multiple sections
There is substantial duplication of text in the Introduction, Methods, Data Analysis, and Discussion. Several paragraphs appear more than once, and the same concepts are repeated with similar wording.
Suggestion: Remove all duplicated content and streamline each section for clarity.

**Response: All duplicated content has been removed from the clean version of the revised manuscript and the respective sections streamlined for clarity.**

2. Introduction lacks focus and is overly long
The Introduction contains multiple narrative threads (e.g., the Ayesha example, global burden statistics, repeated MCID rationale). This affects the flow and dilutes the study objective.
Suggestion: Condense the Introduction into four clear paragraphs:

Stroke burden and importance of mobility

Role and relevance of the 2MWT

Lack of MCID evidence, especially in LMICs

Precise study aim

**Response: The Introduction section has been streamlined for clarity as recommended above.**

3. Methods section needs clearer organization

The Methods are too lengthy, and several elements (center selection, interventions, statistical procedures) appear twice.
Suggestion: Reorganize under standardized subheadings:

Study Design

Setting and Centers

Participants

Interventions

Outcome Measures

Data Collection

Statistical Analysis

This will greatly improve readability.

**Response: The Methods section has been streamlined for clarity as recommended above.**
4. Inconsistencies in tables and figures

There are repeated figure numbers, tables described more than once, and some figures lacking captions.

Suggestion: Ensure each table/figure appears only once and is clearly numbered and labeled.
A standard structure could be:

Table 1: Centers

Table 2: Baseline characteristics

Table 3: Pre–post outcomes

Table 4: Subgroup MCID

Table 5: Comparison with other populations
**Response: All figures and tables captions have been correctly improved in light of the recommendation above, all figures and tables have been numbered correctly and appropriately.**

5. Discussion contains unnecessary repetition

The Discussion restates parts of the Introduction and Results and includes multiple general statements.

Suggestion: Structure into four concise paragraphs:

Key findings

Comparison with existing literature

Strengths and limitations

Implications and future research

**Response: The Discussion section has been streamlined for clarity as recommended above and redundant statements have been deleted.**

6. Language and style issues

Some sentences are long, conversational, or informal. Tense usage is inconsistent, and clarity can be improved.

Suggestion: Edit for concise, scientific language with consistent past tense.

**Response: The clean version of the revised manuscript has been improved in light of the recommendation above.**

7. Duplication of ethical and administrative content

Funding, competing interests, and ethics approval statements appear in more than one section.

Suggestion: Present this information only once in the designated section.

**Response: The ethics statement has been placed within in its designated location within the methods section and its duplication in other locations deleted.**

Minor Comments

Include a STROBE/CONSORT-style flow diagram.

**Response: We have included a STROBE flow diagram with a title “Fig 1: Participant Flow Chart” in the clean version of the revised manuscript**

Clarify your missing data handling approach.

**Response: Missing data occurred in 8% of cases, primarily due to participant dropout, and were addressed using multiple imputation with five iterations to minimize bias. (Line 182-184 of the clean copy of the revised manuscript). A sensitivity analysis using complete-case analysis confirmed that MCID estimates remained consistent (32–34 meters) supporting the robustness of the imputation approach. (Line 181-182 of the clean copy of the revised manuscript)**

State whether assessors were trained or calibrated for inter-rater reliability.

**Response: Assessor variability was minimized through standardized training (Line 325 of the Discussion section in the clean copy of the revised manuscript).**

Provide the formula used for sample size calculation.

**Response: We have provided a formula for sample size determination under the participant section of the Methods and further clarified how we reached sample size of 150 participants. (Line 109-126 of the clean copy of the revised manuscript)**

Briefly explain the clinical meaning of the 33-m MCID (e.g., change in gait speed or functional relevance).

**Response: The 33 meter minimally clinically important difference refers to the smallest change in performance on a 6 meter walk test that a patient or clinician would perceive as a meaningful change in patients’ functional capabilities.**

Overall Recommendation

This is a valuable study with strong methodological foundations and clear clinical implications. However, substantial revisions—particularly reducing repetition, reorganizing structure, and tightening the writing—are needed to improve clarity and prepare the manuscript for publication.

**Response: Many thanks for your valuable insights in improving our manuscript and all comments have been put into consideration while making the necessary revisions to our manuscript.**
