# Supplementary material for: Validating the 2-minute walk test MCID for subacute stroke patients: A Pakistani multicenter cohort analysis
Source: PLoS One. 2026 Apr 15;21(4):e0347056. doi: 10.1371/journal.pone.0347056 (PMC13082714; doi:10.1371/journal.pone.0347056)
Supplement: Attachment — Submitted filename: Dear Editor.docx [file pone.0347056.s006.docx]

Dear Editor,

I have reviewed the manuscript entitled **“Validating the 2-Minute Walk Test MCID for Subacute Stroke Patients: A Pakistani Multicenter Cohort Analysis.”** The study addresses an important and clinically relevant topic in stroke rehabilitation, particularly the establishment of a minimally clinically important difference (MCID) for the 2-Minute Walk Test, which is widely used in resource-limited settings.

The manuscript demonstrates several strengths, including a multicenter design, a relatively large sample for an MCID study, and the use of an anchor-based approach supported by distribution-based analyses. The reported MCID appears clinically plausible and may offer meaningful guidance for rehabilitation professionals.

However, I have significant concerns that warrant **major revision** prior to potential acceptance. Most notably, there is a lack of clarity and internal consistency regarding the sample size calculation versus the final analyzed sample, inconsistencies in reported baseline values and timelines, and substantial redundancy across sections. In addition, language quality, formatting errors, and conflicting data availability statements detract from the overall scientific rigor and presentation.

Importantly, these concerns appear **correctable** and do not fundamentally undermine the study’s concept or potential contribution. With careful revision focusing on methodological transparency, numerical consistency, reduced repetition, and professional language editing, the manuscript could become suitable for publication.

**Recommendation:** **Major Revision**

Thank you for the opportunity to review this manuscript.

Sincerely,
Reviewer
