# Supplementary material for: Validating the 2-minute walk test MCID for subacute stroke patients: A Pakistani multicenter cohort analysis
Source: PLoS One. 2026 Apr 15;21(4):e0347056. doi: 10.1371/journal.pone.0347056 (PMC13082714; doi:10.1371/journal.pone.0347056)
Supplement: Attachment — Submitted filename: Dear Authors.docx [file pone.0347056.s007.docx]

Dear Authors,

Thank you for the opportunity to review your manuscript entitled “Validating the 2-Minute Walk Test MCID for Subacute Stroke Patients: A Pakistani Multicenter Cohort Analysis.” The study addresses a clinically meaningful and timely topic in neurorehabilitation, particularly relevant for resource-limited settings. Establishing a minimally clinically important difference (MCID) for the 2-Minute Walk Test (2MWT) in subacute stroke patients is of clear practical value for clinicians and researchers.

The multicenter design, relatively large sample size, and use of both anchor-based and distribution-based approaches are notable strengths. However, several methodological, reporting, and presentation issues should be addressed to improve the scientific clarity and overall quality of the manuscript.

Major Comments

1. Sample Size Justification
   - The manuscript reports an initial sample size calculation of *n = 431*, while the final analyzed sample consists of *150 participants*.
   - The rationale for this discrepancy is insufficiently explained and may raise concerns regarding statistical power.

Recommendation:
Please clearly clarify whether the calculated sample refers to the screened population or revise the sample size justification using MCID-specific considerations appropriate for anchor-based analyses.

2. Methodological Consistency
   - There are inconsistencies in the reporting of baseline 2MWT values (e.g., 32.8 m vs. 62.5 m) across sections.
   - The Methods section mentions “paired t-tests every three months,” which does not align with the reported 6–8 week intervention period.

Recommendation:
Carefully review and harmonize all numerical data and timelines throughout the manuscript to ensure consistency.

3. Redundancy Across Sections
   - The importance and robustness of the 33-meter MCID are repeatedly stated in the Introduction, Results, and Discussion.
   - Several paragraphs reiterate similar interpretations without adding new insights.

Recommendation:
Streamline the narrative to reduce repetition and improve readability, particularly in the Introduction and Discussion sections.

4. Data Availability Statement
   - The manuscript contains conflicting statements regarding data accessibility (public availability vs. availability upon request).

Recommendation:
Please ensure compliance with journal data-sharing policies by providing a single, clear, and accurate data availability statement.

5. Language and Formatting
   - The manuscript contains numerous typographical errors, formatting artifacts (e.g., tracked changes), and grammatical issues.
   - These issues detract from the clarity and professionalism of the submission.

Recommendation:
A thorough language edit is strongly recommended prior to resubmission.

Minor Comments

1. Introduction
   - The Introduction would benefit from a more focused background and avoidance of anecdotal or narrative examples.
   - Consider sharpening the rationale for selecting the ABC_Gait subscale as the primary anchor.
2. Outcome Measures
   - Please clarify whether assessors were blinded to baseline measurements and intervention details.
3. Figures and Tables
   - Some figures appear redundant with tabulated results.
   - Consider reducing the number of figures to improve conciseness.
4. Discussion
   - The comparative discussion with other populations (e.g., COPD, MS) is appropriate and informative.
   - A brief paragraph translating the 33-meter MCID into direct clinical application would further strengthen this section.

Conclusion

In summary, this study has the potential to make a valuable contribution to the stroke rehabilitation literature. Addressing the points outlined above—particularly those related to sample size justification, data consistency, and manuscript clarity—will significantly enhance the quality and impact of the work.

I encourage the authors to revise the manuscript accordingly and look forward to reviewing a revised version.

Sincerely,
Reviewer
