# Supplementary material for: Validating the 2-minute walk test MCID for subacute stroke patients: A Pakistani multicenter cohort analysis
Source: PLoS One. 2026 Apr 15;21(4):e0347056. doi: 10.1371/journal.pone.0347056 (PMC13082714; doi:10.1371/journal.pone.0347056)
Supplement: Attachment — Submitted filename: Response_to_Reviewers_auresp_2.docx [file pone.0347056.s008.docx]

**Manuscript reference:** [PONE-D-25-43400R1] - [EMID:28fdc4b985fb05dd]

**Decision:** Major Revision

**Title:** Validating the 2-minute walk test MCID for subacute stroke patients: A Pakistani multicenter cohort analysis.

**PLOS ONE,**

***Dear Editor,***

We appreciate your oversight of our manuscript and the opportunity to address the insightful comments from the respective reviewers. We extend our sincere gratitude to the reviewers for their valuable feedback which is aimed at improving our manuscript.

Please find below our point-by-point response to all the issues raised by the reviewers. Our responses below list the reviewers’ comments followed by a description of the necessary amendments we the authors have made in bold.

The page numbers of the responses to reviewer comments correspond to the clean version of the revised manuscript.

We look forward to hearing from you at your earliest convenience.

Kindly,

Edward Muteesasira.

**Reviewer #2**

***Dear Authors, Thank you for the opportunity to review your manuscript entitled “Validating the 2-Minute Walk Test MCID for Subacute Stroke Patients: A Pakistani Multicenter Cohort Analysis.” The study addresses a clinically meaningful and timely topic in neurorehabilitation, particularly relevant for resource-limited settings. Establishing a minimally clinically important difference (MCID) for the 2-Minute Walk Test (2MWT) in subacute stroke patients is of clear practical value for clinicians and researchers.
The multicenter design, relatively large sample size, and use of both anchor-based and distribution-based approaches are notable strengths. However, several methodological, reporting, and presentation issues should be addressed to improve the scientific clarity and overall quality of the manuscript.*

Major Comments**
1. Sample Size Justification
o The manuscript reports an initial sample size calculation of n = 431, while the final analyzed sample consists of 150 participants.
o The rationale for this discrepancy is insufficiently explained and may raise concerns regarding statistical power.
Recommendation:
Please clearly clarify whether the calculated sample refers to the screened population or revise the sample size justification using MCID-specific considerations appropriate for anchor-based analyses.
**Response: The calculated sample size refers to the screened population. We have added a short statement under the sample size determination section of the methodology that says “The study population sample size to be screened was calculated using…….” (Line 109-110) of the clean version of the revised manuscript**

2. Methodological Consistency
¬ There are inconsistencies in the reporting of baseline 2MWT values (e.g., 36.2m vs. 62.5 m) across sections.

**Response: We recorded 2MWT values at Baseline(pre-intervention) and post intervention(6-8 weeks). Therefore 62.5m refers to the 2MWT value at baseline while 36.2m refers to the 2MWT value post intervention. This is reported on Table 3 of the clean version of the revised manuscript.**
¬ The Methods section mentions “paired t-tests every three months,” which does not align with the reported 6–8-week intervention period.

**Response: We have modified this statement to “Paired t-tests were performed every three weeks on 2MWT, ABC, ABC_Gait, and MBI scores”. The incorrect reporting was a typo error. (Line 182 of the clean version of the revised manuscript)**

Recommendation:
Carefully review and harmonize all numerical data and timelines throughout the manuscript to ensure consistency.
**Response: All numerical data and timelines have been reviewed and harmonized throughout the manuscript.**

3. Redundancy Across Sections
¬ The importance and robustness of the 33-meter MCID are repeatedly stated in the Introduction, Results, and Discussion.
¬ Several paragraphs reiterate similar interpretations without adding new insights.
Recommendation:
Streamline the narrative to reduce repetition and improve readability, particularly in the Introduction and Discussion sections.

**Response: All redundant paragraphs in the introduction, results and discussion section repeatedly stating the robustness of the 33-meter MCID have been deleted from these sections. The introduction and discussion sections have been further refined to improve readability and conciseness.**
4. Data Availability Statement
¬ The manuscript contains conflicting statements regarding data accessibility (public availability vs. availability upon request).
Recommendation:
Please ensure compliance with journal data-sharing policies by providing a single, clear, and accurate data availability statement.

**Response: we have refined our data availability statement to “Data sets are available upon reasonable request from the corresponding author” (Line 337 of the clean version of the revised manuscript)**
5. Language and Formatting
¬ The manuscript contains numerous typographical errors, formatting artifacts (e.g., tracked changes), and grammatical issues.
¬ These issues detract from the clarity and professionalism of the submission.
Recommendation:
A thorough language edit is strongly recommended prior to resubmission.

**Response: We have addressed the numerous typographical errors, formatting artifacts and grammatical issues as well as the clean version of the revised manuscript has undergone a thorough language edit to improve readability and scholarly power of the manuscript.**

**Minor Comments**
1. Introduction
¬ The Introduction would benefit from a more focused background and avoidance of anecdotal or narrative examples.

**Response: The introduction has been streamlined in light of this recommendation**
¬ Consider sharpening the rationale for selecting the ABC_Gait subscale as the primary anchor.

**Response: The rationale for selecting the ABC_Gait subscale as the primary anchor has been clearly stated and sharpened and is reported on lines 178-180 of the clean version of the revised manuscript as,”** **A 10% improvement in ABC_Gait was selected as the anchor based on prior studies indicating this threshold as clinically meaningful for patient-reported balance confidence in stroke populations”**

**Response:**
2. Outcome Measures
¬ Please clarify whether assessors were blinded to baseline measurements and intervention details.

**Response: We have added a short statement on line 151 of the clean version of the revised manuscript stating,”** **Assessors were blinded to baseline measurements and intervention details”.**
3. Figures and Tables
¬ Some figures appear redundant with tabulated results.
¬ Consider reducing the number of figures to improve conciseness.

**Response: We have removed redundant figures in order to improve conciseness.**
4. Discussion
¬ The comparative discussion with other populations (e.g., COPD, MS) is appropriate and informative.
¬ A brief paragraph translating the 33-meter MCID into direct clinical application would further strengthen this section.

**Response: We have added a brief paragraph on Line 303-304 of the clean version of the revised manuscript stating,”** **A 33-meter increase in the 2MWT indicates meaningful mobility gains, enabling clinicians to prioritize intensive, tailored interventions to enhance independence in subacute stroke patients.”**

Conclusion
In summary, this study has the potential to make a valuable contribution to the stroke rehabilitation literature. Addressing the points outlined above—particularly those related to sample size justification, data consistency, and manuscript clarity—will significantly enhance the quality and impact of the work.
I encourage the authors to revise the manuscript accordingly and look forward to reviewing a revised version.
**Response: Many thanks dear reviewer for your insightful recommendations aimed at improving the readability and conciseness of our manuscript.**
