# Supplementary material for: Validating the 2-minute walk test MCID for subacute stroke patients: A Pakistani multicenter cohort analysis
Source: PLoS One. 2026 Apr 15;21(4):e0347056. doi: 10.1371/journal.pone.0347056 (PMC13082714; doi:10.1371/journal.pone.0347056)
Supplement: Attachment — Submitted filename: Overall Impression.docx [file pone.0347056.s009.docx]

**Overall Impression**

This is an important and clinically useful study. Determining the MCID of the 2-Minute Walk Test (2MWT) in subacute stroke patients is highly relevant, especially for rehabilitation settings with limited resources. The multicenter design and use of both anchor-based and distribution-based methods are strengths.

However, some methodological clarifications, statistical explanations, and language corrections are needed before publication.

**Major Comments**

**1. Sample Size Calculation Needs Clarification**

The sample size calculation is based on a formula for comparing proportions, but this study focuses on determining MCID using ROC analysis.

- Please explain why this formula was used.
- Clarify whether 150 participants were enough for MCID estimation.
- If possible, provide additional justification or a post-hoc power explanation.

**2. Inconsistent Baseline 2MWT Values**

There is a difference in the reported baseline values:

- One section reports baseline as 32.8 meters.
- Table 3 reports 62.5 ± 38.4 meters.

Please correct this inconsistency to avoid confusion.

**3. Justification of 10% ABC_Gait Anchor**

You used a 10% improvement in ABC_Gait as the anchor.

- Please explain clearly why 10% was chosen.
- Is this cut-off validated in subacute stroke patients?
- Consider briefly explaining why GPE was not used as the primary anchor.

**4. Differences Between Centres**

Some centres used robotic gait training while others did not.

- Was the centre effect adjusted for in the analysis?
- Please clarify whether this variability could influence the MCID.

**5. Missing Data**

You mention that 8% of data were imputed.

- Which variables were imputed?
- What method of multiple imputation was used?
- Why were five iterations considered sufficient?

**6. Statistical Reporting**

- Please report confidence intervals for key changes.
- Clarify whether normality was tested before using paired t-tests.
- Explain clearly what the logistic regression odds ratio (OR = 1.15) represents.

**7. Figures Need Improvement**

- The ROC curve appears almost diagonal despite a high AUC (0.89). Please check the figure.
- The scatter plot looks overly linear. Confirm that it reflects actual raw data.
- Improve overall figure resolution and labeling.

**8. Data Availability Statement Is Inconsistent**

In one section, it states that all data are available in the manuscript.
In another section, it says data are available upon request.

Please revise to match PLOS ONE data policy.

**9. Generalizability**

The study includes only subacute stroke patients.

- Please discuss more clearly whether results apply to chronic stroke.
- Expand slightly on rural vs. urban rehabilitation differences.

**Minor Comments**

**Language and Grammar**

There are several small grammar errors throughout the manuscript. A careful language edit is recommended.

**Consistency**

- Use either 2MWT or 2WMT consistently.
- Keep formatting consistent for ABC_Gait.
- Use either “33 m” or “33 meters” consistently.

**Tables and Repetition**

- Some subgroup results are repeated in multiple sections. Remove repetition.
- Table formatting can be simplified.

**Strengths**

- Multicentre design
- Clinically meaningful outcome
- Strong correlation findings
- Practical value for low-resource settings

**Recommendation**

**Major Revision**

The study is valuable and relevant, but clarification and careful editing are needed before publication.
