# Supplementary material for: Validating the 2-minute walk test MCID for subacute stroke patients: A Pakistani multicenter cohort analysis
Source: PLoS One. 2026 Apr 15;21(4):e0347056. doi: 10.1371/journal.pone.0347056 (PMC13082714; doi:10.1371/journal.pone.0347056)
Supplement: Attachment — Submitted filename: Response_to_Reviewers_auresp_3.docx [file pone.0347056.s010.docx]

**Manuscript reference:** [PONE-D-25-43400R2] - [EMID:e1fc739dfaa58053]

**Decision:** Revision Required

**Title:** Validating the 2-minute walk test MCID for subacute stroke patients: A Pakistani multicenter cohort analysis.

**PLOS ONE,**

***Dear Editor,***

We appreciate your oversight of our manuscript and the opportunity to address the insightful comments from the respective reviewers. We extend our sincere gratitude to the reviewers for their valuable feedback which is aimed at improving our manuscript.

Please find below our point-by-point response to all the issues raised by the reviewers. Our responses below list the reviewers’ comments followed by a description of the necessary amendments we the authors have made in bold.

The page numbers of the responses to reviewer comments correspond to the clean version of the revised manuscript.

We look forward to hearing from you at your earliest convenience.

Kindly,

Edward Muteesasira.

**Academic Editor’s comments**

Thank you for your assistance. After careful evaluation, I agree with the reviewer that the following concerns should be addressed by the authors prior to acceptance:

A significant concern was the reduction from a calculated n=431 to a final n=150. The authors clarified that 431 represented the number of patients screened for eligibility, while 150 met the criteria and completed the study. Nonetheless, the authors' decision to utilize only 150 participants instead of 431 to mitigate potential statistical power issues remains ambiguous.

**Response: We thank the reviewer for this observation. We have revised the sample size determination section of the methodology to clarify that the 431 individuals represented the initial screening pool and not the actual sample size, our actual sample size “n” is the 150 participants that met all inclusion/exclusion criteria and completed the study protocol. Our study’s initial power analysis was based on a target of 150 participants. With an alpha of 0.05 and an expected effect size of 0.5 therefore a sample of 150 provided a statistical power of 80%. Thus, the final sample size was sufficient to test our primary hypothesis without affecting the statistical power of the study. In conclusion,** **the reduction from 431 to 150 was dictated by strict clinical eligibility criteria and participant retention rather than an arbitrary decision. This is presented in Fig 1,the CONSORT Flow Diagram to transparently detail the specific reasons for exclusion at each stage. Line 108-127 of the clean version of the revised manuscript.**

The manuscript provides a justified, literature-based rationale for the 10% ABC_Gait anchor, but a slightly more detailed explanation would further strengthen its clarity and persuasiveness.

**Response: We have expanded this rationale in the Methods section of the clean version of the revised manuscript. The 10% threshold was selected as it represents a change that exceeds the typical measurement error and is recognized in literature as a clinically meaningful "patient-perceived" improvement in balance confidence for stroke survivors. This threshold ensures that the anchor reflects a shift that is perceptible to the patient rather than minor day-to-day fluctuations. Line 179-181 of the clean version of the revised manuscript.**

The manuscript fails to adequately justify the potential impact of intervention variability or center effects. The absence of analysis or discourse on this subject constitutes a limitation that must be rectified for a more comprehensive interpretation of the results.

**Response: We acknowledge this as an important factor. To mitigate center effects, we utilized a standardized therapist training manual and conducted biweekly audits across all seven sites. Furthermore, our post-hoc subgroup analysis comparing centers with and without robotic gait training and showed no significant difference in 2MWT MCID (p=0.21). We have now added a paragraph in the "Limitations" section discussing how standardized goals across diverse centers actually enhance the generalizability of the 2MWT MCID to real-world Pakistani clinical settings. Line 305-310 of the clean version of the revised manuscript.**

The authors should address the comment, "Some figures, particularly the ROC curve and scatter plot, should be carefully reviewed to ensure they accurately reflect the data and are clearly presented."

**Response: Fig 4 and Fig 5 have been revised to include explicit axis labels, the AUC value, the 33m cutoff point, and a linear regression line to visually represent the correlation (r=0.68) as requested.**

The following points should be considered:

The ROC Curve (Fig 4) should clearly display the AUC, the cutoff point, sensitivity, and specificity.
The axes should be labeled ("1-Specificity," “Sensitivity”), and the cutoff (33 meters) should be indicated if possible.
The figure should match the numbers stated in the Results section (AUC = 0.89, sensitivity = 87%, specificity = 82%).

**Response: We have revised Fig 4 (ROC Curve). The axes are now explicitly labeled "Sensitivity" and "1-Specificity." The figure clearly displays the AUC of 0.89, with the 33-meter cutoff point indicated, showing 87% sensitivity and 82% specificity, matching the text in the results.**

The scatter plot (Fig 5) should illustrate the true relationship between the 2MWT and ABC_Gait change scores.
The reported correlation (r = 0.68) should be visually evident.
Axes must be labeled with correct units and variable names, and any outliers should be identifiable or explained.

**Response:** **Fig 5 (Scatter Plot) has been updated to show the linear relationship between 2MWT change scores (meters) and ABC_Gait change scores. The axes are now clearly labeled with units. Statistical analysis confirmed no significant outliers (p > 0.05), which is now stated in the results.**

Finally, the manuscript would benefit from careful language editing to correct minor grammatical issues and improve overall clarity and flow.

**Response:** **The clean version of the revised manuscript has undergone a thorough review to correct minor grammatical errors, such as ensuring consistency in grammar and refining transitions between the results and discussion sections.**

**Reviewer #2**

**Overall Impression**

This is an important and clinically useful study. Determining the MCID of the 2-Minute Walk Test (2MWT) in subacute stroke patients is highly relevant, especially for rehabilitation settings with limited resources. The multicenter design and use of both anchor-based and distribution-based methods are strengths.

However, some methodological clarifications, statistical explanations, and language corrections are needed before publication.

**Major Comments**

**1. Sample Size Calculation Needs Clarification**

The sample size calculation is based on a formula for comparing proportions, but this study focuses on determining MCID using ROC analysis.

- Please explain why this formula was used.
- Clarify whether 150 participants were enough for MCID estimation.
- If possible, provide additional justification or a post-hoc power explanation.

**Response:** **The initial sample size was calculated using a comparison of proportions to ensure sufficient power to distinguish between those that underwent “manual gait training” from the “robotic gait training” group, this provided a robust cohort of 150 participants, exceeding the stability requirements for the subsequent ROC analysis used to determine the MCID.**

**2. Inconsistent Baseline 2MWT Values**

There is a difference in the reported baseline values:

- One section reports baseline as 32.8 meters.
- Table 3 reports 62.5 ± 38.4 meters.

Please correct this inconsistency to avoid confusion.

**Response: We have removed all inconsistencies in reporting of results to avoid confusion.**

**3. Justification of 10% ABC_Gait Anchor**

You used a 10% improvement in ABC_Gait as the anchor.

- Please explain clearly why 10% was chosen.
- Is this cut-off validated in subacute stroke patients?

**Response: A 10% improvement in ABC_Gait was selected as the anchor based on prior studies indicating this threshold as clinically meaningful for patient-reported balance confidence in subacute stroke populations. Yes, this cut off is validated in subacute stroke patients (Bowman et al,)**

- Consider briefly explaining why GPE was not used as the primary anchor.

**Response: While recognizing the fact that the Global Perceived Effect (GPE) is a patient centered outcome measure, its methodological limitations particularly its susceptibility to recall bias hindered its use as the primary anchor for this study. Our study assessed participants at two points; T0: Baseline (pre-intervention) and T1: Post-intervention (6-8 weeks), we would have faced significant limitations with recall bias especially in the follow-up phase (post-intervention assessment). In order to minimize the risk of recall bias, we utilized the ABC_Gait subscale (≥10% improvement) as the anchor.**

**4. Differences Between Centers**

Some centers used robotic gait training while others did not.

- Was the center effect adjusted for in the analysis?
- Please clarify whether this variability could influence the MCID.

**Response:** **To explore the impact of intervention variability, a post-hoc subgroup analysis was conducted to compare 2MWT improvements between centers with and without robotic gait training. No significant differences were observed (p = 0.21), suggesting that the 33-meter MCID is robust across varying intervention modalities.**

**5. Missing Data**

You mention that 8% of data were imputed.

- Which variables were imputed?

**Response: Participant sociodemographic characteristics and primary outcome measures were imputed.**

- What method of multiple imputation was used

**Response: multiple imputation**

- Why were five iterations considered sufficient?

**Response: Missing data occurred in 8% of cases, primarily due to participant dropout, and were addressed using** **multiple imputation with five iterations to minimize bias. A sensitivity analysis using complete-case analysis confirmed that MCID estimates remained consistent (32–34 meters) supporting the robustness of the imputation approach.**

**6. Statistical Reporting**

- Please report confidence intervals for key changes.

**Response: Confidence intervals for key changes have been reported in the clean version of the revised manuscript.**

- Clarify whether normality was tested before using paired t-tests.

**Response: Yes,** **normality was tested before using paired t-tests.**

- Explain clearly what the logistic regression odds ratio (OR = 1.15) represents.

**Response: This has been stated in the clean version of the revised manuscript.**

**7. Figures Need Improvement**

- The ROC curve appears almost diagonal despite a high AUC (0.89). Please check the figure.
- The scatter plot looks overly linear. Confirm that it reflects actual raw data.
- Improve overall figure resolution and labeling.

**Response: Response: We have revised Fig 4 (ROC Curve). The axes are now explicitly labeled "Sensitivity" and "1-Specificity." The figure clearly displays the AUC of 0.89, with the 33-meter cutoff point indicated, showing 87% sensitivity and 82% specificity, matching the text in the results. Fig 5 (Scatter Plot) has been updated to show the linear relationship between 2MWT change scores (meters) and ABC_Gait change scores. The axes are now clearly labeled with units. Statistical analysis confirmed no significant outliers (p > 0.05), which is now stated in the results.**

**8. Data Availability Statement Is Inconsistent**

In one section, it states that all data are available in the manuscript.
In another section, it says data are available upon request.

Please revise to match PLOS ONE data policy.

**Response: we have refined our data availability statement to ”The minimal dataset required to replicate our study findings is available at Figshare:** [**https://figshare.com/articles/figure/_b_Determining_the_Minimally_Clinically_Important_Difference_of_the_2-Minute_Walk_Test_in_Individuals_with_Subacute_Stroke_A_Multicenter_Cohort_Study_b_/29484728**](https://figshare.com/articles/figure/_b_Determining_the_Minimally_Clinically_Important_Difference_of_the_2-Minute_Walk_Test_in_Individuals_with_Subacute_Stroke_A_Multicenter_Cohort_Study_b_/29484728)**”**

**9. Generalizability**

The study includes only subacute stroke patients.

- Please discuss more clearly whether results apply to chronic stroke.

**Response: We reported this as a limitation for our study and stated that validation in chronic stroke populations is needed to extend its applicability.**

- Expand slightly on rural vs. urban rehabilitation differences.

**Response: We have reported in the discussion section that “Within Pakistan, urban centers often provide better access to rehabilitation facilities and technologies (e.g., robotic gait training), while rural areas face barriers like transportation and limited therapist availability. This disparity may affect the applicability of the 33 meters MCID, as rural patients may have less frequent therapy sessions.”**

**Minor Comments**

**Language and Grammar**

There are several small grammar errors throughout the manuscript. A careful language edit is recommended.

**Response: The clean version of the revised manuscript has undergone a thorough review to correct minor grammatical errors, such as ensuring consistency in grammar and refining transitions between the results and discussion sections.**

**Consistency**

- Use either 2MWT or 2WMT consistently.
- Keep formatting consistent for ABC_Gait.
- Use either “33 m” or “33 meters” consistently.

**Response: We have put this into consideration while making the required amendment to ensure consistency.**

**Tables and Repetition**

- Some subgroup results are repeated in multiple sections. Remove repetition.

**Response: Repetitions have been removed in the clean version of the revised manuscript.**

- Table formatting can be simplified.

**Response: Table formatting has been simplified.**

**Recommendation**

**Major Revision**

The study is valuable and relevant, but clarification and careful editing are needed before publication.
